# Pixel-level metal blackbody microcavities via hierarchical laser writing

**DOI:** 10.1126/sciadv.adu0608

**Published:** 2025-02-28

**Authors:** Chong-Kuong Ng, Tianle Chen, Bing-Feng Ju, Yuan-Liu Chen, Yungui Ma

**Affiliations:** ^1^ State Key Lab of Modern Optical Instrumentation, Centre for Optical and Electromagnetic Research, College of Optical Science and Engineering; International Research Center (Haining) for Advanced Photonics, Zhejiang University, Hangzhou 310058, China.; ^2^ State Key Lab of Fluid Power and Mechatronic Systems, ZJU-Hangzhou Global Scientific and Technological Innovation Center, Zhejiang University, Hangzhou 310058, China.

## Abstract

Conventional blackbody cavities, known for their near-unity broadband omnidirectional emissivity (absorptivity), are however constrained by their large volume (e.g., >10^4^ cm^3^), imposing crucial restrictions on integration with existing devices. Here, we introduce the concept of metal blackbody microcavities, comprising thousands of microscale periodic pores created on metals, demonstrating excellent emissivity across visible and infrared (IR) ranges (exceeding 0.94 on average from 0.25 to 20 μm). In the long-wavelength IR (8 to 14 μm) region, near-unity emissivity was successfully achieved by 100-μm-deep metal microcavities with ultralow structural aspect ratios, facilitated by laser-textured multiscale surface morphologies that substantially enhance the light-trapping capabilities. Our findings demonstrate that microcavity-based patterns can produce local emissivity, tunable radiative intensity gradients, wide-angle feasibility, and high-temperature resistance, thereby enabling diverse applications in thermal IR displays such as thermal illusion, IR encryption, and grayscale thermal imaging. Notably, these blackbody microcavities are applicable to various metals, presenting considerable potential for use in extreme environments.

## Teaser

Compact blackbody microcavities exhibit high emissivity and stability, enabling advanced thermal IR applications.

## INTRODUCTION

**Fig. 1.**
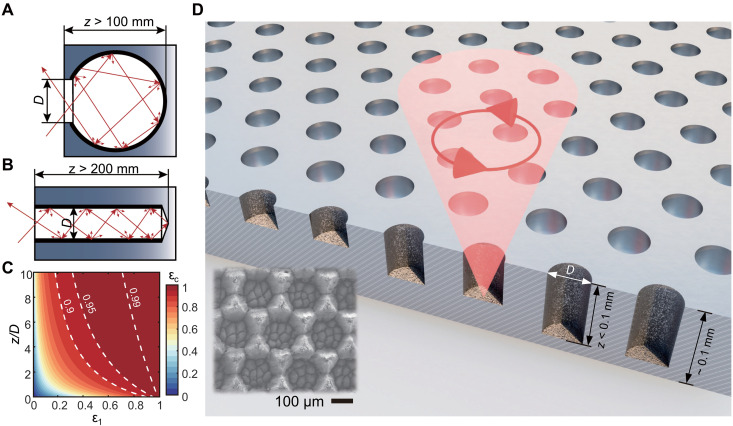
Concept of metal blackbody microcavities. Traditional metal blackbodies, such as (**A**) spherical cavities and (**B**) cylindrical cavities, operate as a light trap, in which incident light (red arrows) undergoes multiple reflections and is finally absorbed. (**C**) Effect of the emissivity ε_1_ of the internal surfaces of the cavity and the aspect ratio *z/D* on the effective emissivity ε_c_ of a cylindrical blackbody cavity. (**D**) Schematic of the metal blackbody microcavities using circularly polarized laser writing. Inset is SEM image of the titanium blackbody microcavities with a constant period *P* = 200 μm and diameters *D* = 190 μm.

Blackbody is an ideal object that can absorb all radiation incident on its surface or emit radiation in reverse. In practice, it can be approximated by a small aperture in a large, bulky chamber with lossy internal surfaces. These so-called blackbody cavities have been widely used as calibration standards for spectral measurements of absorptivity or emissivity (*1*). Modeled in geometrical optics, the local effective emissivity ε_c_ of these cavities can be evaluated as (*2*, *3*)

$$\varepsilon_{c}={\left[1+\left(\frac{1}{\varepsilon_{1}}-1\right)F\right]}^{-1}$$ (1)

where ε_1_ is the emissivity of the internal diffusive surfaces of the cavity and *F* represents the view factor from the cavity interior to the aperture, as a function of the aspect ratio *z/D* (*z* is the depth of the cavity and *D* is the diameter of the aperture, as detailed in note S1). For instance, to reach ε_c_ > 0.95, when *D* = 50 mm, the depth is *z* > 100 mm for the spherical cavity (Fig. 1A), and *z* > 200 mm for the cylindrical cavity (Fig. 1B). However, the substantial size and bulkiness of conventional macroscale blackbodies substantially limit their mobility, integration, and compatibility with existing devices. In addition, there also exist challenges in drilling light-trapping holes with manipulated diffuse surfaces to achieve higher emissivity ε_1_ while maintaining the aspect ratio (Fig. 1C). Therefore, the pursuit of miniaturized blackbodies without compromising design and performance holds great promise for practical applications. Although carbon nanotubes (CNTs) with a thickness of several hundred micrometers meet these criteria and achieve unmatched emissivity (> 0.99 on average) over an ultrawide spectral range from 0.2 to 200 μm wavelength (*4*, *5*), they are easily degraded by mechanical wear or exposure to harsh environments (*6*).

Metallic materials dominate modern devices and components for daily and industrial purposes, typically machined through turning, milling, boring, and grinding (*7*, *8*). Millimeter-sized compact blackbodies, such as triangular V-grooves (*9*), periodic pyramids (*10*, *11*), and multiple-cell hexagonal (honeycomb) structures (*1*, *12*), have been developed to modify the emissivity of metallic materials. However, constrained by mechanical processes, compact blackbodies with dimensions below 1 mm are rarely discussed, which motivates our work. On the other hand, most of the existing thermal metamaterials are fabricated using lithography-based techniques or composites, which pose challenges in compatibility with rough metallic surfaces (*13*–*15*). In contrast, femtosecond (fs) laser ablation stands out as highly efficient, flexible, versatile, and extensively used in diverse materials, offering a multiscale resolution spanning from centimeters to nanometers (*16*–*18*). Regardless of surface roughness, highly flexible fs laser writing not only supports the fabrication of large-scale miniature blackbodies on metal surface, but also allows the customization of complex infrared (IR) patterns using advanced graphics algorithms (*19*). Planar microblackbodies fabricated by fs lasers, capable of seamless integration with metal-based devices, may revolutionize current thermal technologies, such as energy harvesting (*20*, *21*), thermal camouflage (*15*, *22*), thermal management (*6*, *23*), radiative cooling (*24*, *25*) and IR sensing (*5*, *26*).

Here, we aim to explore the feasibility of manufacturing shallow metal blackbody microcavities with exceptional broadband emissivity using an advanced hierarchical fs-laser writing technique (Fig. 1D) directly on metals, demonstrating the potential of these structures for the development of innovative thermal devices and technologies. Compared with conventional composite (*13*, *22*) or metamaterial absorption coatings (*13*, *26*), densely distributed metal blackbody microcavities provide notable advantages in terms of stability and lifespan in extreme conditions (*21*), as well as greater flexibility in engineering the local emissivity. We show that the emissivity at local regions (~100 μm) can be adjusted across a wide range (2.5 to 20 μm), from approximately 0.1 to 0.94, by controlling the geometric dimensions of the microcavities. Specifically, we discovered that the highly rough, diffuse surfaces with unique, gradient morphologies produced by fs laser ablation could greatly reduce the aspect ratio *z/D* required for microcavities, enabling the creation of ultrathin, compact blackbodies. Moreover, thermal IR radiation performs distinct advantages in scenarios involving darkness, solar glare, and bad weather, stimulating keen interest in scalable perception and encryption in IR wavelengths (*27*). By leveraging the spatial radiative distribution (*28*, *29*), we demonstrate the promising future of microcavities in encoding thermal IR information, showcasing remarkable capabilities such as multigradient tunable intensity, wide-angle feasibility, and high-temperature resistance. This has led to the development of a digital thermal IR printing strategy for the high-resolution grayscale IR pictures.

## RESULTS

### Design and fabrication of hexagonal microcavities

**Fig. 2.**
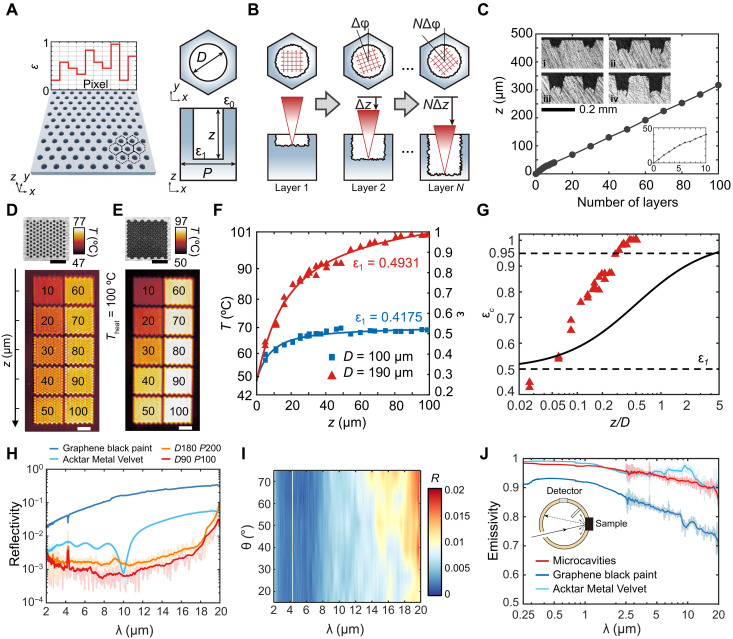
Design and fabrication of blackbody microcavities. (**A**) Pixel-level metal blackbody microcavities for programming local emissivity, where each hexagonal periodic meta-atom consists of a single microcavity. (**B**) Schematics of the hierarchical laser writing process with increasing depth *z* from left to right, including in-plane scanning (top, red lines represent scanning paths of laser) and subsequent *z*-moving (bottom) procedures. (**C**) Dependence of laser-engraved depths *z* on the number of layers, with a zoomed-in view in the bottom right. Insets (i to iv) are cross-sectional views of microcavities with *D* = 100 μm. (**D** and **E**) IR images of microcavities with different depths *z* from 10 to 100 μm in steps of 10 μm, a constant period *P* = 200 μm, and diameters *D* = 100 μm for (D) and *D* = 190 μm for (E). Visible images are shown on top and detailed in fig. S11. Scale bars, 1 mm. (**F**) Dependence of radiative temperatures and depths *z* of microcavities with different diameters. (**G**) Comparison of cavity emissivity ε_c_ for microcavities (*D* = 190 μm, red dots) and conventional cylindrical cavities (ε_1_ = 0.5, solid line). (**H**) Specular reflectivity of microcavities with *D* = 180 μm, *P* = 200 μm and *D* = 90 μm, *P* = 100 μm, compared with two blackbody coatings. (**I**) Incident angle dependence of specular reflectivity of microcavities. (**J**) Emissivity (ε = 1 − ρ) of large-scale microcavities and the blackbody coatings. Inset shows the schematic of the hemispherical reflectivity ρ measurement using an integrating sphere. Dark lines in (H) and (J) denote the filtered curves, using a moving average of 40 neighboring points.

Assuming Lambertian surfaces with thermal and optical homogeneity, surface morphology considerably affects the spectral emissivity of metal (*3*), such as boosting the emissivity ε_c_ of microcavities. In addition, the hexagonal periodic unit, incorporating a single cylindrical micropore (ε_c_ term) and the flat surface (ε_0_ term), is defined as the meta-atom (Fig. 2A). On the basis of the principle of synthetic apertures, its expression can be derived as

$$\varepsilon_{\text{meta}}=f\varepsilon_{c}+\left(1-f\right)\varepsilon_{0}$$ (2)

where ε_c_ is the cavity emissivity, ε_0_ is the flat surface emissivity, and *f* is the filling factor of microcavities, representing the area ratio between the microcavity and the unit cell. This formula not only introduces extra degrees of freedom (e.g., *D*, *P*, and *z*) for adjusting the local emissivity, but also helps determine unknown parameters (e.g., ε_1_). Given the rough texture of metallic bulk materials, fabricating or integrating functional optical microstructures on metallic devices presents formidable challenges (*16*). To address this, we propose height-tunable micro/nanofabrication for the metal blackbody microcavities using hierarchical fs laser writing (Fig. 1D). Circular polarization is used to avoid the asymmetric ablation caused by polarization (*30*). Hierarchical laser writing is achieved through a two-step process repeatedly (Fig. 2B, from left to right). First, orthogonal lateral scans remove material layer by layer, with each layer offset of Δφ in each layer. Next, the stage’s *z*-axis motion is precisely programmed to control laser spot feeding at a fixed interval of Δ*z*, preparing for the subsequent lateral scans. A linear relationship between the number of scanning layers and the depth *z* is indicated in Fig. 2C, with the processing parameters of table S1. Note that the cavity emissivity ε_c_ of the microcavities may still follow geometric optics principles (Eq. 1), ensuring effective absorption of IR radiation, because their size exceeds several wavelengths [e.g., *D* > 10 λ_p_, as shown in the scanning electron microscopy (SEM) of Fig. 1D, where λ_p_ = *b/T* is the wavelength peak of the thermal radiation, *b* = 2898 μm·K by Wien’s law].

The metallic blackbody microcavities (fig. S13) were successfully fabricated using a fs laser with a high resolution of 13 μm, favorably enhancing broadband emissivity in the long-wavelength infrared (LWIR) regime. Unlike conventional macrocavities that require coatings, these microcavities are distinguished by their fs laser fabrication, which introduces unique characteristics, such as precise machining quality and inherent rough microstructures (fig. S14). Compared to continuous or nanosecond lasers, femtosecond lasers minimize the heat-affected zone and prevent thermal damage, ensuring superior machining precision due to a much shorter thermal diffusion length relative to the optical skin depth (*31*). In addition, thermal accumulation during laser scanning produces a mixture of plasma, vapor, and nanodroplets that subsequently resolidify to form black microstructures (*32*). These laser-induced black microstructures, featuring pronounced roughness, intrinsically exhibit high surface emissivity (*20*, *33*, *34*), which can be tailored by adjusting laser processing parameters such as pulse energy (e.g., fig. S9), hatching density, and scanning velocity. As laser-material ablation in air often involves oxidation (e.g., fig. S14), the formation of metal oxides further increases LWIR absorption. The laser-induced roughness and oxide layers simultaneously lead to a diffuse, lossy surface, eliminating the need for blackbody coatings. In essence, the multiscale surface morphologies produced by the fs laser, including periodic microcavities (~100 μm) and laser-induced microstructures (on the order of micrometers), allow for single-step, coating-free fabrication of miniature blackbodies on metals. Furthermore, by engineering the geometric dimensions and spatial distribution of microcavities, we can independently control local emissivity at the pixel level, unlocking the potential for encoding complex IR information on metallic surfaces.

### Pixel-level local emissivity of metal blackbody microcavities

Next, the thermal IR properties of metal blackbody microcavities are experimentally verified. Because characterizing an individual microcavity poses challenges, we instead measured the IR radiative temperature of periodic microcavity arrays using an LWIR camera. To control the thermal emissivity and radiative temperatures, we fabricated the blackbody microcavities with varying depths *z* on titanium sheets, while maintaining the periods and diameters constant (e.g., Fig. 2, D and E), as detailed in fig. S11. Adjusting the microcavity depths allows for tunable radiative temperatures in IR images, even when the heating temperature is fixed at *T*_heat_ = 100°C. No distinct visual difference is observed in their visible images, as if the IR information was encrypted. The cross-sectional views and SEM images of these microstructures can be found in figs. S12 and S13, respectively. On the basis of Stefan-Boltzmann law, the emissivity of the object can be calculated from the temperatures as

$$\varepsilon =\frac{J_{\text{Obj}}}{J_{\text{BB}}}=\frac{\left(T_{\text{rad}}^{4}-T_{\text{amb}}^{4}\right)}{\left(T_{\text{sur}}^{4}-T_{\text{amb}}^{4}\right)}$$ (3)

The surface temperature was *T*_sur_ = 101°C, the ambient temperature was *T*_amb_ = 20°C, and the radiative temperature *T*_rad_ was measured from the IR images (e.g., fig. S11). To eliminate fluctuations in IR temperature readings, a static calibration was conducted as described in fig. S26. Note that the temperature in Eq. 3 must be converted to the kelvin scale, K = °C + 273.15. Furthermore, by deriving Eqs. 1 and 2, the nonlinear fitting model for the emissivity of meta-atoms can be formulated as

$$\varepsilon =p_{0}+p_{1}{\left(1+p_{2}F\right)}^{-1}\approx p_{0}+p_{1}\left[1-p_{2}F+{\left(p_{2}F\right)}^{2}-{\left(p_{2}F\right)}^{3}\right]$$ (4)

where *p_i_* (*i* = 0, 1, 2) are the fitting coefficients. From this model, we can determine the emissivity ε_0_ of the flat surface by the *y*-intercept of fitting curves, the emissivity ε_1_ of the internal surfaces of microcavities by *p*_2_ = 1/ε_1_ − 1, and the estimated filling factor by *p*_1_ = f^*^. For instance, ε_0_ = 0.27 and ε_1_ = 0.49 for *D* = 190 μm, and ε_0_ = 0.27 and ε_1_ = 0.42 for *D* = 100 μm, as shown in Fig. 2F. Note that the estimated filling factor f^*^ slightly differs from the area-ratio value *f*, where f^*^ ≠ *f*. This difference introduces a gain (or attenuation) in the terms of ε_c_, leading to the broadening (or narrowing) of the tunable range of emissivity, Δε = ε_max_ − ε_min_. For example, Δε reaches 0.74 with f^*^ = 0.91 > *f* = 0.82 for *D* = 190 μm, whereas Δε is only 0.10 with f* = 0.13 < *f* = 0.23 for *D* = 100 μm. We therefore conducted a regression analysis to determine the filling factor f^*^, and emissivity ε_0_ and ε_1_, where ε_1_ is typically challenging to measure using conventional techniques. By separating the constant term (e.g., ε_0_ for flat surfaces) using the coefficients of fitting curves, the cavity emissivity ε_c_ of microcavities can be further obtained for comparison with the ε_c_ of conventional cavities directly (Fig. 2G). Even in the geometry-wave optics transition regime (e.g., *z* = 10 to 100 μm), the cavity emissivity ε_c_ gradually increases with the depth *z*, following the blackbody principle (Eq. 1). Unexpectedly, to achieve blackbody-like emissivity (e.g., ε_c_ > 0.95), an ultralow aspect ratio of *z/D* > 0.31 for *D* = 190 μm microcavities is sufficient, compared to the conventional requirement of *z/D* > 4.5 for macrocavities, which is more than 14 times greater. We observed that the minimal thickness of an ultrathin blackbody microcavity can approach wavelength-scale dimensions (e.g., *z* > 60 μm = 4 λ at 15 μm). The laser-oxidized rough surfaces, treated as gradient dielectrics based on effective medium theory (*35*), exhibit strong wave effects. These microcavities, with such surface, therefore contribute to geometry-wave optical coupling effects, including scattering, surface plasmon polaritons (SPPs), and enhanced absorption (*33*, *36*).

Moreover, we characterized the optical properties of the titanium blackbody microcavities in the wavelength ranges from the visible to IR regime. Figure 2H demonstrates an ultralow specular reflectivity *R* that reaches 10^−3^ in the IR regime (2 to 20 μm) for two microcavities. The average reflectivity is *R* = 0.001 for *D* = 90 μm, *P* = 100 μm and *R* = 0.002 for *D* = 180 μm, *P* = 200 μm, respectively. The reflectivity of microcavities even surpasses that of commercial blackbody coatings by over an order of magnitude (e.g., *R* = 0.008 for Acktar Metal Velvet, *R* = 0.077 for graphene black paint). These properties are also confirmed by the mid-wavelength infrared (MWIR) and LWIR images, as illustrated in fig. S15. In the following, we selected the former structures (*D* = 90 μm, *P* = 100 μm) as the optimal blackbody microcavities. Figure 2I shows that the optimal blackbody microcavities maintain the low reflectivity of <0.01 in oblique incidence angle even up to 70°. Using an integrating-sphere spectrometer, as shown in Fig. 2J, large-scale (25 mm by 25 mm) blackbody microcavities on the titanium sheet (fig. S16A) performed an excellent hemispherical emissivity in both VIS-NIR (ε_VIS-NIR_ = 0.970 across 0.25 to 2.5 μm) and IR ranges (ε_IR_ = 0.939 across 2.5 to 20 μm). This performance matches that of Acktar Metal Velvet (ε_VIS-NIR_ = 0.973 and ε_IR_ = 0.948), while surpassing that of graphene black paint (ε_VIS-NIR_ = 0.91 and ε_IR_ = 0.86). The ultralow specular reflectivity and considerable hemispherical emissivity of microcavities demonstrate extremely strong scattering and absorptivity on the pure metal substrates, radically modifying the optical characteristic of the original titanium sheets, which have an average emissivity ε_IR_ = 0.32 (fig. S10). Nevertheless, a slight decrease in hemispherical emissivity (ε < 0.9) at the longer wavelength of 20 μm likely suggests that the limited dimensions of the microcavities restrict certain wave optics–based modes (fig. S25), thereby degrading the light-trapping ability. To demonstrate the general applicability of our techniques, we experimentally demonstrate that all the blackbody microcavities fabricated on various metal materials (e.g., Ti, Al, Cu, Fe, Ni, and W, as shown in fig. S17) exhibit near-perfect blackbody performance. The excellent emissivity in broadband wavelengths can effectively absorb the solar spectrum, thereby facilitating solar thermal applications, such as water sanitation and thermoelectric generation (*20*, *33*).

### Depth-encoded blackbody microcavities for thermal IR encryption

**Fig. 3.**
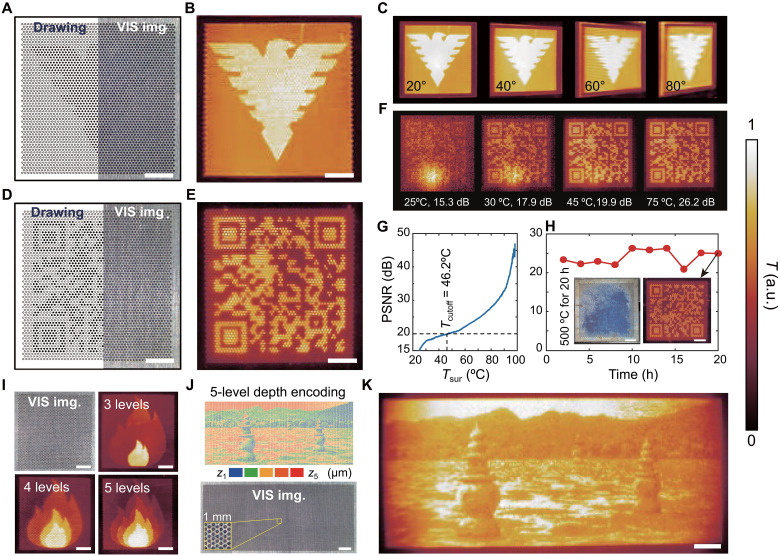
Depth-driven thermal IR encryption. (**A**) Drawing, visible (VIS) image and (**B**) thermal IR image of Zhejiang University logo with a size of 10 mm by 10 mm. (**C**) IR images of Zhejiang University logo at four tilted angles. (**D**) Drawing, visible image, and (**E**) IR image of the QR code with a size of 10 mm by 10 mm. Drawings of (A) and (D) use dark circles for deep microcavities and light circles for shallow microcavities to encode the patterns. (**F**) Thermal IR images of the QR code with their PSNR at different heating temperatures. (**G**) Effect of surface temperature on PSNR of IR images, using the IR image at 100°C as a reference. (**H**) Testing high-temperature (*T* = 500°C) resistance: effect of duration time on PSNR of IR images at 100°C. Insets are visible and IR images of the QR code sample after it has been heated to 500°C for 20 hours. Both (G) and (H) are using the QR code sample to test PSNR of IR images. (**I**) Multilevel temperature tunability of encrypted fire patterns, all of which show a similar visible image (e.g., in the top left). (**J**) Drawing, visible image (including a 1-mm-sized zoomed-in view in the bottom left), and (**K**) thermal IR image of Hangzhou West Lake with a size of 25 mm by 10 mm using pixelated five-level depth encoding. All those patterns were created by microcavities with *D* = 100 μm and *P* = 200 μm. Scale bars, 2 mm. h, hours.

In the previous section, we indicated that the radiative temperature of microcavities can be freely tuned by the cavity depth, with no substantial difference in their visible images (e.g., fig. S11). This effect inspires an IR encrypting strategy by depth-encoded blackbody microcavities. We created the IR encrypted pattern of the logo of Zhejiang University using dual-depth microcavities, showcasing hidden depth information in Fig. 3A but a distinct IR image in Fig. 3B. To explore the mechanism behind this encryption, the simulation of an IR encrypted letter “A” with dual-level depths (fig. S6) suggests that the nonuniformity of surface temperature arises from the distinct thermal radiosity on the pattern with deep micropores, where the localized radiation effect plays a crucial role. As illustrated in fig. S18, an LWIR camera mounted on manual stages was used to capture the IR image at oblique angles, while a thermocouple was used to measure the surface temperature of the heating plane. By using this setup, the wide-angle feasibility of microcavities is confirmed by IR encrypted images of the logo of Zhejiang University, clearly viewing at tilted angles from 20° to 80° (Fig. 3C). Similarly, the IR encoded pattern of the quick response (QR) code with an alternative dual-depth drawing (Fig. 3D) displays detectable IR information (Fig. 3E).

In addition to investigating the temperature dependence of the IR image, we experimentally tested the effect of surface temperature on the IR image quality (Fig. 3F) and calculated the variation of the peak signal-to-noise ratio (PSNR) of the IR images in various temperatures (Fig. 3G). The cutoff temperature of the QR code is *T*_cutoff_ = 46.2°C when PSNR > 20 dB, leading to unnoticeable noise in IR images at high temperatures (e.g., > 45°C in Fig. 3F). The IR temperature readings of specific regions often reflect coupling effects between ambient radiation and the radiation emitted by the object itself, particularly in low emissivity and temperature (close to ambient levels), where ambient radiation becomes critical. Because the radiative energy is proportional to the fourth power of temperature, we commonly heat the samples at 100°C to minimize the effects of ambient radiation. We proved that the IR encrypted patterns of metal microcavities can be operated in high-temperature (*T* = 500°C) environments with long-term stability (Fig. 3H). The IR images remain unchanged with a PSNR of more than 20 dB despite gradually serious oxidation after 20 hours of annealing at 500°C (fig. S21). The variation of the PSNR of IR images may be attributed to mismatch errors and misfocusing issues. Microcavities created on high temperature–resistant metals, such as nickel-based and cobalt-based alloys, can operate reliably in extreme temperature environments (*T* > 800°C), where radiative heat predominates, to facilitate IR display capabilities.

Furthermore, beyond the dual-level radiative temperature of microcavities, the IR encrypted patterns of fires can display at most five-level temperatures while these patterns hide their depth information (Fig. 3I). The corresponding drawings and visible images of these multigradient fires are illustrated in fig. S19. Through programming five-level depth in each local area, we printed the IR encrypted picture of Hangzhou West Lake using the Floyd-Steinberg dithering algorithm (Fig. 3, J and K). By introducing noises or errors, dithered approximation of the image can display 8-bit and even more color, in conditions of limitation of colors (*37*). For instance, even though the same five-level palette is used, a dithered image gives a better representation of the original without color banding in large regions, compared to the truncated image, as shown in fig. S20. However, when programming the depth of microcavities, few grayscale levels (at most five levels) in local radiative temperature (local emissivity) lead to a substantial restriction for displaying lossless 8-bit grayscale images in the IR range. This limitation arises from the considerable initial emissivity ε_0_ = 0.273 of rough titanium sheets and the low depth resolution (> 2.5 μm as detailed in table S1) of the hierarchical laser writing.

### Ultrahigh-grayscale and high-resolution thermal image printing

**Fig. 4.**
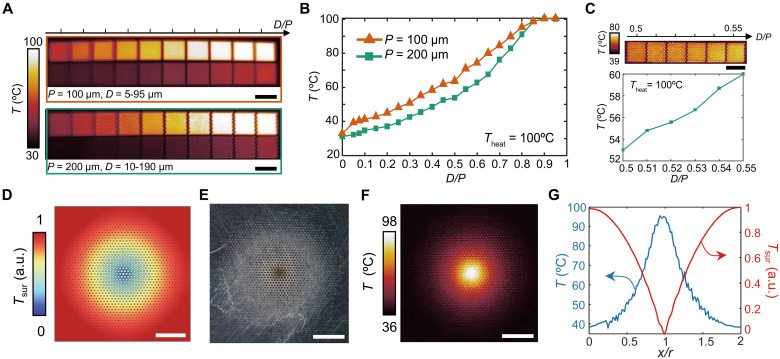
Local emissivity engineering. (**A**) Thermal IR images of microcavities with difference diameters *D* for *P* = 100 μm (top) and *P* = 200 μm (bottom). (**B** and **C**) Dependence of radiative temperatures *T* and the length ratio *D/P* of microcavities measured in (A) and the inset of (C). (**D**) Simulation of surface temperature of the gradient-radiative circle. (**E**) Visible image and (**F**) thermal IR image of the gradient-radiative circle. (D) to (F) are using the same pattern with a constant period *P* = 200 μm. (**G**) Radial distributions of measured radiative temperatures in (F) (blue line) and simulated surface temperatures in (D) (red line). Scale bars, 2 mm.

In contrast to encoding depths, by engineering the widths of microcavities, we introduce an alternative strategy to control the local emissivity of the metal surface over a wide tunable range, from approximately 0.1 to more than 0.9. When the emissivity of rough titanium sheets exceeds 0.3, polished aluminum sheets with a low initial emissivity ε_0_ < 0.1 were used to facilitate a greater tunability in radiative temperature (fig. S2B). The emissivity in IR range was measured using Fourier transform infrared spectroscopy (FTIR) with an integrating sphere (fig. S10). Therefore, we selected aluminum substrates to avoid the effect of initial roughness. To illustrate the tunability of radiative temperature across an extensive range of gradients, periodic microcavities with various diameters *D* are presented with quasi-continuous gradients (20 levels) of radiative temperature (Fig. 4A). A nonlinear relationship between radiative temperature *T* and the filling factor (quantified as the length ratio *D/P*) is observed in both microcavities with two periods (*P* = 100 μm and *P* = 200 μm), as shown in Fig. 4B. In addition, we fabricated microcavities on titanium substrates, as shown in fig. S23, demonstrating that the tunable temperature range of microcavities on titanium (rough substrates) is narrower than those on aluminum (smooth substrates).

To obtain a higher resolution in radiative temperature tunability, microcavities with a narrow range of *D/P* from 0.5 to 0.55 can be separated into six grayscales to achieve a minor gradient of radiative temperature of Δ*T* = 1.40°C when *P* = 200 μm (Fig. 4C). Theoretically, this allows for up to 50 levels of temperature gradient over a range of approximately 70°C (e.g., Fig. 4B). Notably, the minimal detectable temperature gradient Δ*T* depends on many factors including the heating temperature, ambient radiation, and the sensitivity of the IR camera. The corresponding visible images of microcavities with various diameters are indicated in fig. S22. To preform quasi-continuous temperature gradients, the gradient-radiative circle was fabricated with variable diameters *D* decreasing linearly and radially while the period *P* remains constant, exhibiting a nonlinear radial distribution of temperature, resembling a Gaussian-beam illusion (Fig. 4, E to G). Simulation of the gradient-radiative circle (Fig. 4D) highlights the fall in surface temperature at the center where IR emissivity is strong, leading to the local radiative heat dissipation. Figure 4G shows the comparison between measured radiative temperatures and simulated surface temperatures.

**Fig. 5.**
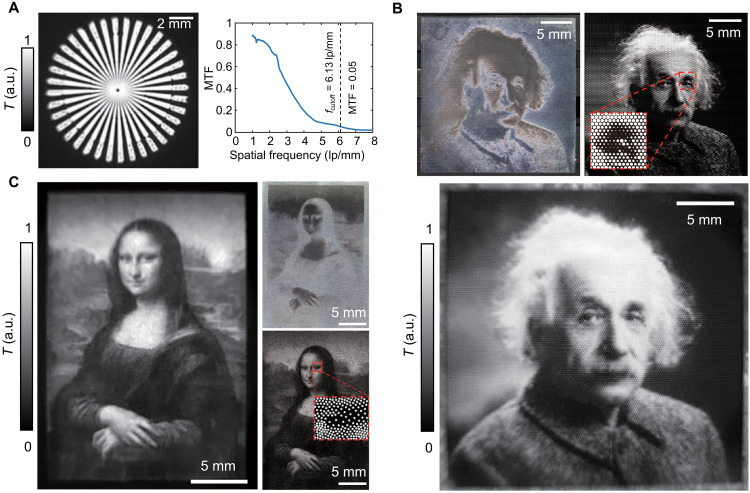
High-resolution thermal image printing on metal. (**A**) Thermal IR image of the resolution star target (Siemens star) and the modulation transfer function (MTF). (**B**) Visible image (top left), drawing (top right), and thermal IR image (bottom) of Albert Einstein, where the microcavities with a constant hexagonal period *P* = 200 μm and various diameters *D* are engraved on a 25 mm by 25 mm region of polished aluminum. (**C**) Visible image (top right), drawing (bottom right), and IR image (left) of Mona Lisa, created by microcavities on a 17 mm by 25 mm area of rough titanium with various periods *P* and a constant diameter *D* = 25 μm using a stippling effect. The visible images represent negative film of the IR images, depending on the current coloring method.

In addition to simple multigradient patterns, we produced intricate IR grayscale pictures with high spatial resolution by laser-engraved metal microcavities, demonstrating the tunability of pixel-level emissivity. First, by adjusting various widths *D* of microcavities, the modulation transfer function (MTF) of the IR camera was calculated from the resolution star target (Fig. 5A), delivering a cutoff spatial frequency of *f*_cutoff_ = 6.13 lp/mm (equal to the minimal linewidth of *w*_cutoff_ = 82 μm) at the resolution limit MTF = 0.05. More details of the resolution star are found in fig. S24. Because the waist radius of the fs-laser spot is approximately 6.44 μm, the microcavities engraved by this laser system, with a minimal diameter of *D* = 13 μm < *w*_cutoff_, fully satisfy the resolution requirements of most commercial IR cameras. Then, the IR grayscale image of Albert Einstein is patterned on polished aluminum sheets, using various diameters *D* on hexagonal periodic (*P* = 200 μm) microcavities, as shown in Fig. 5B. Inspired by pointillism artworks, we also printed an IR grayscale image of Mona Lisa using a stippling effect (*19*), showcasing a quasi-continuous grayscale radiative temperature in Fig. 5C. This image uses thousands of microcavities with constant diameters *D* = 25 μm and random intervals, created on rough titanium sheets. The stippled images (e.g., Fig. 5C) seem to have a natural and smooth gradient transition, rendering it more suitable for artistic creations [e.g., augmented reality (*29*)], whereas pixelated images (e.g., Fig. 5B) are more appropriate for digital applications [e.g., point-to-point data storage (*38*)] due to their inherent periodicity. These two examples additionally demonstrate that the robustness offered by the blackbody microcavities empowers the IR grayscales printing in diverse materials regardless of high topographic relief and roughness. Thus, we open up a digital printing strategy for IR information, presenting exciting opportunities for the integration of IR devices across many applications.

## DISCUSSION

We have reported metal blackbody microcavities consisting of thousands of periodic micropores on metals, with the minimal thickness of only four times the wavelength. The ultrathin blackbody microcavities are applicable in various metals regardless of the surface roughness, achieving a lossy Lambertian characteristic with outstanding hemispherical emissivity (ε > 0.94) and ultralow specular reflectivity (*R* < 0.004 at incidence angles of more than 70°) across a broadband wavelength range of 0.25 to 20 μm. The versatility offered by hierarchical fs laser writing empowers the fabrication of microcavities that can individually specify local emissivity in each pixel to harness the radiative temperature spatially. By rationally programming geometric dimensions of microcavities, we can encode local emissivity as encrypted IR information and print grayscale IR pictures with high spatial resolution. Given that no commercial electronics memory device can reliably operate at temperatures above 300°C (*39*), our thermal microstructures with the extreme temperature resistance have the potential to serve as a robust alternative for long-term data storage in harsh environments, such as aerospace and energy exploration. The metal blackbody microcavities with symmetry are limited to modulating radiation in a single aspect, which is intensity. Nevertheless, advanced thermal photonics generally involve the extended manipulation of angular, spectral, and polarization characteristics through broken symmetries in materials or geometries (*40*–*43*). In further works, we plan to develop asymmetric microstructures to explore more sophisticated functionalities and intriguing physical phenomena.

## MATERIALS AND METHODS

### Laser fabrication system

A femtosecond fiber laser (Femto-10, Huaray) with a central wavelength of 1035 nm, a repetition rate of 1 MHz, a pulse width of 400 fs, and a maximum power of 10 W was used to fabricate the metals. The output power of this laser was manipulated by an external analog voltage signal. The beam diameter was expanded using a beam expander with a magnification of 3×. The expanded laser passed through the polarization modulation elements consisting of a polarizing beam splitter (PBS), a half–wave plate, and a quarter–wave plate, and then was then relayed to the galvanometer (IntelliSCAN III 10, SCANLAB GmbH) equipping an *f*-θ lens (f-65T-1030-1080, TK opto-electric) with a focal distance of 65 mm for focusing into the samples. Typically, the waist radius of the fs laser was evaluated as ω_0_ = 6.44 μm using Liu’s method (*44*). Micropositioning was achieved using a motorized XYZ stage (KWC06020, Surugaseiki) that is capable of providing a microscale step with a resolution of 0.05 μm in a 20-mm travel range for each axis. In addition, it is necessary to remove the debris using a high-pressure air jet to prevent the blockage of microcavities during laser processing. The overall laser fabrication system is shown in fig. S8.

### Sample preparation

Metals including titanium (Ti-6Al-4V, Grade 5), aluminum (5052), copper (C11000), electro-galvanized steel (SECC), nickel (Ni200), and tungsten (W1, >99.95% pure) were obtained from Taobao. Titanium and aluminum alloys were selected as the main materials for creating the blackbody microcavities. The root mean square roughness was characterized as *R*_q_ = 2.4 nm for aluminum and *R*_q_ = 269 nm for titanium using white-light interferometry. Three commercial blackbody coatings, including graphene black paint (*45*), Acktar Metal Velvet (*46*), and Musou black (*47*), were purchased and applied to metal substrates for comparison. The laser-processing parameters are provided in table S1. All laser-treated samples were ultrasonic cleaned with ethanol for 10 min and blow-dried with high-pressure air.

### Numerical simulations

Heat transfer simulations in this work were conducted with a finite-element method solver in COMSOL Multiphysics. Heat transfer was coupled to the surface-to-surface radiation to calculate the effective emissivity and the surface temperature of the microcavities. In addition, two-dimensional (2D) radiative flux in free space was simulated using the module of “radiation in participating media.” Detailed settings and discussions can be found in note S2.

### Characterization

The top and sectional views of the microcavities were characterized using optical microscopy (LV100D, Nikon). SEM images and energy-dispersive x-ray spectra were obtained using a field-emission scanning electron microscope (SU8010, Hitachi). The 3D morphology and surface roughness were characterized using white-light interferometry (NewView 8200 Series, ZYGO). The specular reflectivity and microscopic emissivity in the IR wavelength range (2 to 20 μm) were measured using 15× Cassegrain objective (numerical aperture = 0.4) in an FTIR (VERTEX 70, Bruker) with an IR microscope (HYPERION 2000, Bruker). The measurement of microscopic emissivity follows the method outlined in (*23*). The angular reflectivity in the IR regime (2 to 20 μm) was characterized by the FTIR with a variable angle reflection accessory (A513, Bruker). The reflectivity spectra were collected from different angles from 13° to 83° manually. A gold mirror was used as a reference to normalize the FTIR reflectivity. The hemispherical reflectivity was characterized in two wavelength ranges: 0.25 to 2.5 μm and 2.5 to 20 μm. The hemispherical reflectivity in the first range was characterized using an ultraviolet (UV)–visible (VIS)–near-infrared (NIR) spectrometer (Cary 5000, Agilent) coupled with an integrating sphere. The hemispherical reflectivity in the second range was characterized via the FTIR equipped with an integrating sphere (A562, Bruker). On the basis of Kirchhoff’s law of radiation, the emissivity was calculated by ε = 1 − *R*, where *R* is the hemispherical reflectivity. LWIR images were captured using an LWIR camera (640 × 512 pixels, TD2067T, Hikmicro) with a vanadium oxide (VO_2_) uncooled sensor detecting wavelengths in 8 to 14 μm. The emissivity of the LWIR camera is set at a constant value of 0.96 for all samples. Notably, if not otherwise specified, IR images were captured at the heating temperature of 100°C using the LWIR camera. A K-type thermocouple was used to measure the surface temperature of the heating plane. MWIR images were collected using an MWIR camera (640 × 512 pixels, ImageIR 8300, Infratec) operating in the wavelength range of 1.5 to 5.5 μm.

## Supplementary Materials

### The PDF file includes:

Supplementary Text Figs. S1 to S26 Tables S1 to S3 Legend for movie S1 References
