# Supplementary material for: Pixel-level metal blackbody microcavities via hierarchical laser writing
Source: Sci Adv. 2025 Feb 28;11(9):eadu0608. doi: 10.1126/sciadv.adu0608 (PMC11870063; doi:10.1126/sciadv.adu0608)
Supplement: Supplementary file 1 — Supplementary Text Figs. S1 to S26 Tables S1 to S3 Legend for movie S1 References [file sciadv.adu0608_sm.pdf]

Science Advances
AAAS

Supplementary Materials for

Pixel-level metal blackbody microcavities via hierarchical laser writing

Chong-Kuong Ng *et al.*

Corresponding author: Yungui Ma, yungui@zju.edu.cn; Yuan-Liu Chen, yuanliuchen@zju.edu.cn

Sci. Adv. **11**, eadu0608 (2025)
DOI: 10.1126/sciadv.adu0608

The PDF file includes:

Supplementary Text
Figs. S1 to S26
Tables S1 to S3
Legend for movie S1
References

Other Supplementary Material for this manuscript includes the following:

Movie S1

Supplementary Text

Note S1. Modeling the effective emissivity of cavities

Traditional blackbody cavities take advantage of the multiple reflections on the diffuse surface of cavities, resulting in enhanced absorption of the incident light (e.g., Fig. 1, A and B). Assuming that the Lambertian reflectivity ρ_1 is uniform over the internal surface of the cavity and obeys Kirchhoff's law and conservation of energy.

$$\varepsilon_1 = \alpha_1 = 1 - \rho_1 \tag{S1}$$

where α_1 is the emissivity and ε_1 is the absorptivity. We consider the incident light with an energy of unity. After irradiating into the cavity and subsequently reflecting on the internal surface, the light is divided into two portions, which are the reflected energy $\rho^{(1)} = \rho_1 F$ for the first reflection and the absorbed energy of α_1. Since the reflected light is partially intercepted by the cavity, the view factor F is defined as a geometric coefficient to quantify the fraction of the intercepted radiation from the cavity to the aperture. Therefore, the first-order emissivity can be written as $\varepsilon^{(1)} = 1-\rho_1 F$, while the total energy left for the subsequent reflections is $1-\rho_1 F-\alpha_1$. Then, the second reflection occurs in the same way, with the reflectivity of $\rho_1^2(1-F)F$. The second-order emissivity is given by $\varepsilon^{(2)} = 1-\rho_1 F-\rho_1^2(1-F)F$. After repeating the reflection and absorption many times, the effective emissivity of the cavity can be calculated by the following converging infinite series (*3*, *28*).

$$\begin{aligned} \varepsilon_c = \varepsilon^{(\infty)} &= 1-\rho_1 F-\rho_1^2(1-F)F-\rho_1^3(1-F)^2F-\rho_1^4(1-F)^3F\ldots \\ &= \frac{1-\rho_1}{1-\rho_1(1-F)} = \left[1+F\left(\frac{1}{\varepsilon_1}-1\right)\right]^{-1} \end{aligned} \tag{S2}$$

where ε_c only represents the effective emissivity in the aperture of the cavities. The view factor of the spherical cavity (F_{SPH}) and cylindrical cavity (F_{CYL}) can be written as follows:

$$F_{SPH} = \left[1+4\left(\frac{z}{D}\right)^2\right]^{-1} \tag{S3}$$

$$F_{CYL} = \left[1+4\frac{z}{D}\right]^{-1} \tag{S4}$$

where z is the depth of the microcavities, D is the diameter of the aperture, and z/D is the aspect ratio of the microcavities. Based on the theory of geometric optics, this model can be extended to our designed microcavities, provided the size of the aperture D of microcavities exceeds the wavelengths. Fig. S1B shows the emissivity equivalent relationship from spherical to cylindrical cavities, primarily attributed to the difference in the view factor F (Fig. S1A).

Note S2. Heat transfer simulation of microcavities

We conduced heat transfer simulation of the blackbody microcavities using finite-element method (FEM) in COMSOL. The simulations include the effective emissivity, the surface temperature, and the thermal radiative fields near microcavities in free space. The titanium alloy Ti-6Al-4V with thermal conductivity κ = 6.7 W/m/K, heat capacity C_p = 526.3 J/kg/K and density ρ = 4.43

g/cm^3 is selected as the material for this simulation (*48*). The upper surface of microcavities subject to an ambient heat convection with a convective coefficient h_{air} = 10 W/m²/K, representing 1-3 m/s light wind situations (*49*), at the room temperature T_{air} = 300 K. Assuming that the emissivity is ε_0 = 0.25 for the upper surface (initial surface) and ε_1 = 0.50 for the internal surface (laser-treated surface) of microcavities, as illustrated in Fig. 2A.

We analyzed the effect of surface morphology and the initial emissivity ε_0 on the effective emissivity ε_{meta} of hexagonal periodic microcavities using FEM simulation. By varying the geometric dimensions (e.g., the diameter *D* and depth *z*) of microcavities, the cylindrical microcavities enable the local emissivity ε_{meta} tunability in a wide range from 0.5 to 0.95 (Fig. S2A). This is further confirmed by the radiative flux of a single microcavity (Fig. S3). The radiative energy increases with the diameter *D* and depth *z* and primarily focuses on the inside of microcavities. However, a higher initial emissivity ε_0 adversely affects the tunable range of radiative temperature (e.g., Fig. S2B) and reduces the number of grayscale gradients in IR images. Moreover, compared to the plane (Fig. S4A), the microcavities (Fig. S4, B and C) can boost thermal emission to the surroundings, thereby facilitating the radiative cooling effect. Compared to microcavities with *D* =100 µm (Fig. S5A), those with *D* =190 µm (Fig. S5B) greatly diminish the temperature on the upper surface. The meta-atom emissivity for the larger microcavities remains greater (e.g., ε_{meta} = 0.72 for *D* =100 µm and ε_{meta} = 0.87 for *D* =190 µm), leading to a more substantial drop in surface temperature. These simulations confirmed that the blackbody microcavities enlarge the surface area and strengthen the heat-convective flow, thereby further reducing the surface temperature.

Note S3. Temperature dependence of the radiative coefficient and detected IR signals

To determine how the temperature effect on thermal radiation, the numerical simulation of the radiative coefficient and detected IR signals is performed based on the theory of heat transfer and Planck's law. In practice, we considered three basic modes of heat transfer, such as conduction, convection, and radiation, which affect surface temperature and radiative signals (*28*). Because of the fabrication of microcavities on thin metal sheets with a thickness of only hundred microns, the surface temperature of microcavities is approximately equal to that of the heating plane. This is attributed to the minimal thermal resistance presented by the thin metal sheet. In contrast to conduction, convection and radiation are generally the predominant modes of heat transfer for the blackbody microcavities. The convective heat flux and radiative heat flux are given by as follows:

$$J_{conv} = h_{air}\left(T_{sur} - T_{air}\right) \tag{S5}$$

$$J_{rad} = \varepsilon_b \sigma\left(T_{sur}^4 - T_{air}^4\right) = h_{rad}\left(T_{sur} - T_{air}\right) \tag{S6}$$

where h_{air} is the convective coefficient, $h_{rad} = \varepsilon_b\sigma(T_{sur}^4 - T_{air}^4)/(T_{sur} - T_{air})$ is the radiative coefficient, ε_b is the emissvity, σ is Stefan–Boltzmann constant, T_{sur} and T_{air} are the surface temperature of microcavities and the ambient temperature, respectively. The radiative coefficient h_{rad} strongly depends on the surface temperature T_{sur}, whereas the convective coefficient h_{air} remains constant. For instance, as shown in Fig. S7A, under the light wind condition with h_{air} = 10 W m^{-2} K^{-1}, convection predominates at low temperatures (e.g., $h_{rad} < h_{air}$ when T_{sur} < 400 K), while radiation is critical at high temperatures (e.g., $h_{rad} > h_{air}$ when T_{sur} > 400 K). The radiative coefficient h_{rad} further increase, becoming substantial for heat transfer at extreme temperatures (e.g., T_{sur} > 800 K).

To determine the temperature dependence of IR images, the ideal detected signals from IR cameras can be integrated over the detection bands (from λ_1 to λ_2) using Planck's law.

$$I_{IR}(T) = \int_{\lambda_1}^{\lambda_2} I_{BB}(\lambda, T) d\lambda \quad \text{(S7)}$$

where I_{BB} is the blackbody spectral irradiance. Fig. S7B shows serveral detected signals of IR cameras operating in different bands, such as short-wave-infrared (SWIR, 0.9-1.7 μm), mid-wave-infrared (MWIR, 3-5 μm) and long-wave-infrared (LWIR, 8-14 μm). Comparing to the other camera, the LWIR camera exhibits the strongest radiative intensity, resulting in being more appropriate to detect the IR signals at low surface temperature (T_{sur} < 632 K) which is easier to obtain.

Note S4. Limitations and efficiency of hierarchical laser writing

In the laser system, the minimum size of microcavities is D = 13 μm, which corresponds to the laser spot size, while the maximum processing area can extend up to 40 mm × 40 mm using a stitching strategy, as shown in Fig. S16A. Despite a scalable dimension in the z-direction, the proposed hierarchical laser writing is time-consuming, with its efficiency influenced by factors including the overall area of patterns, number of layers, and laser scanning parameters. The processing efficiency for microcavities is relatively low compared with conventional laser marking, due to multiple repeated scans in each layer. For instance, using depth resolution Δz = 5 μm and scanning velocity v = 1000 mm/s, patterning microcavities (D = 90 μm, P = 100 μm, z = 100 μm) on a 25 mm × 25 mm region required 20 layers of repeated scans, resulting in a total processing time of 146 minutes. The corresponding processing time per unit area was calculated to be 14 s/mm^2. Additionally, large-scale fabrication of microcavities currently faces several challenges, including such as extended processing time, accumulation of material debris, thermal drift effects of the laser spot and thermal deformation of the sample.

Note S5. Dispersion analysis of metal microcavities

We noted that the metal microcavities can be treated as an ideal cylindrical metal waveguide with infinite extend in z-direction, as shown in Fig. S25A. According to Maxwell's equation, the electromagnetic fields along z-axis can be solved for TE and TM polarized lights, respectively. Generally, we converted the Cartesian coordinate system (x, y, z) to cylindrical coordinates (r, φ, z) for this problem using $r^2 = x^2+y^2$ and $\tan(\varphi) = y/x$.

First, for TE waves with $E_z = 0$, by solving the Maxwell's equation, the electric field in z-direction can be written as

$$H_z = H_0 J_m(\beta_c r) \cos m\varphi e^{-i\beta_z z} \quad \text{(S8)}$$

where H_0 is an arbitrary constant. $J_m(x)$ is the Bessel functions of the first kind. β_z is the propagation wave vector, $\beta_c = \sqrt{\beta_x^2 + \beta_y^2}$ is the in-plane wave vector.

The boundary condition at $r = a$, by solving $\left.\frac{\partial H_z}{\partial r}\right|_{r=a} = 0$, we get

$$\beta_c = \frac{u'_{mn}}{a} \tag{S9}$$

where u'_{mn} is the root of $J'_m(x) = \frac{\partial J_m(x)}{\partial r}$, m is a positive integer, representing the orders of $J_m(x)$, n is the nth root of $J'_m(x)$. Similarly, for TM waves with $H_z = 0$, by the Maxwell's equation, the electric field in z-direction can be written as:

$$E_z = E_0 J_m(\beta_c r)\cos(m\varphi)e^{-i\beta_z z} \tag{S10}$$

where E_0 is an arbitrary constant. The boundary condition at $r = a$, by solving $E_z = 0$, we get

$$\beta_c = \frac{u_{mn}}{a} \tag{S11}$$

where u_{mn} is the nth root of $J_m(x)$. Therefore, the dispersion relation of cylindrical metal waveguide is formulated as

$$\beta_z = \sqrt{\beta^2 - \beta_c^2} \tag{S12}$$

where $\beta = n_0\omega/c$ is the propagation constant and $\omega = 2\pi f$ is the angular frequency. As the incident wave propagates in z-direction, we must have $\beta > \beta_c$ for stable propagation. The cutoff wave vector is calculated as

$$\beta_c = \frac{\omega}{\lambda_c} = \frac{u'_{11}}{a} \tag{S13}$$

In all modes, including both TE_{mn} and TM_{mn} modes, the minimum value of the can be found in the TE_{11} mode, where $u'_{11} = 1.841$. Therefore, the cutoff wavelength is $\lambda_c = 3.41a$. We plotted the dispersion curves of the cylindrical metal waveguide with various radius a = 2, 5, 20 µm , as shown in Fig. S25B. As the radius a increases, more waveguide modes above the cut-off frequency can be supported, leading to a transition from discrete to quasi-continuous frequency coverage. According to the geometry, waveguide modes must propagate stably within chamber, thereby possibly being absorbed. For instance, if a = 2 µm, then λ_c = 6.82 µm and the LWIR wavelengths (8-14µm) absolutely are not presented in this cylinder. Correspondingly, the microcavities with a diameter $D = 2a$ = 4 µm are unable to absorb the wavelengths $\lambda > \lambda_c$ = 6.82 µm. In contrast, we fabricated the blackbody microcavities with $D = 2a$ = 90 µm and calculated the ideal cutoff wavelength of λ_c = 153.45 µm, thereby reasonably achieving an ultra-broadband emissivity from 0.25 to 20 µm.

Additionally, we conducted the number of both TE and TM modes at the wavelength λ > 3 µm while growing the radius a (Fig. S25C). Assuming an ideal blackbody is placed at the output port of the cylindrical waveguide, only the supported waveguide modes will be absorbed. Consequently, the more waveguide modes existed, the higher absorptivity is.

Note S6. Estimation of the modulation transfer function (MTF)

Generally, the modulation transfer function (MTF) is measured from photographs of the resolution test chart to qualify the de facto standard quality of a lens or imaging system (*50, 51*). Alternatively, the MTF can also be computed from the point spread function (PSF). The high resolution of laser writing enables printing the resolution star target (Siemens star) with 36 bars over 360° in a

polished aluminum sheet for IR cameras. As shown in Fig. S24A, the microcavities with various diameters and a constant ratio of diameter to period D/P = 0.95 are utilized to fill the bars of the Siemens star. The center and outer diameters of the star are 0.286 mm and 12 mm, respectively. The maximum resolution at the center circle is f = 40 lp/mm (1016DPI), while the minimum resolution at the outer edge is f = 0.955 lp/mm. In the Siemens star, the spatial frequency $f = N_p/(2\pi r)$ decreases from the center to the outer and is related to the radius r and the number of line pairs N_p = 36. The corresponding line width can be written as $w = 2\pi r/(2N_p)$. We captured the IR image of the fabricated Siemens star placed on a heating plane at 100 °C (Fig. S24B). According to the definition (*50*, *51*), the MTF can be calculated from the contrast ratio of the IR image of the Siemens star.

$$\mathrm{MTF}(f) = \frac{I_{\max} - I_{\min}}{I_{\max} + I_{\min}} \tag{S14}$$

where I_{max} and I_{min} represent the maximum and the minimum values of the intensity of the IR image. The IR intensity (radiative temperature) profile at a specified frequency can be extracted to calculate the MTF. For instance, as shown in Fig. S24C, MTF = 0.05 at f = 6 lp/mm, while MTF = 0.44 at f = 3 lp/mm. Eventually, the MTF at all frequencies is given out, as shown in Fig. 5A.

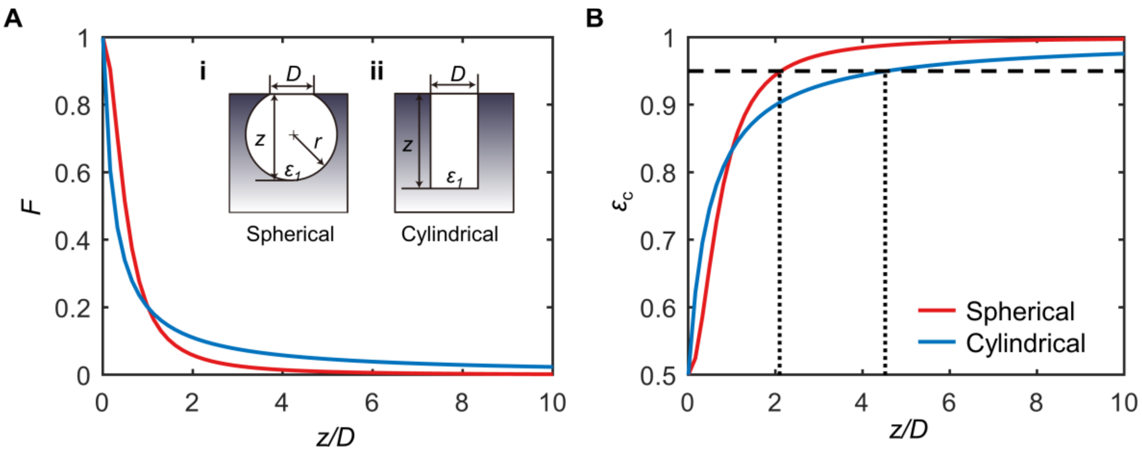

Fig. S1. Equivalent relationship from spherical cavity to cylindrical cavity with ε_1 = 0.5. Effect of the aspect ratio *z/D* of microcavities on (**A**) the view factor *F* and (**B**) the local effective emissivity ε_c. Insets are the schematics of (i) spherical and (ii) cylindrical cavities.

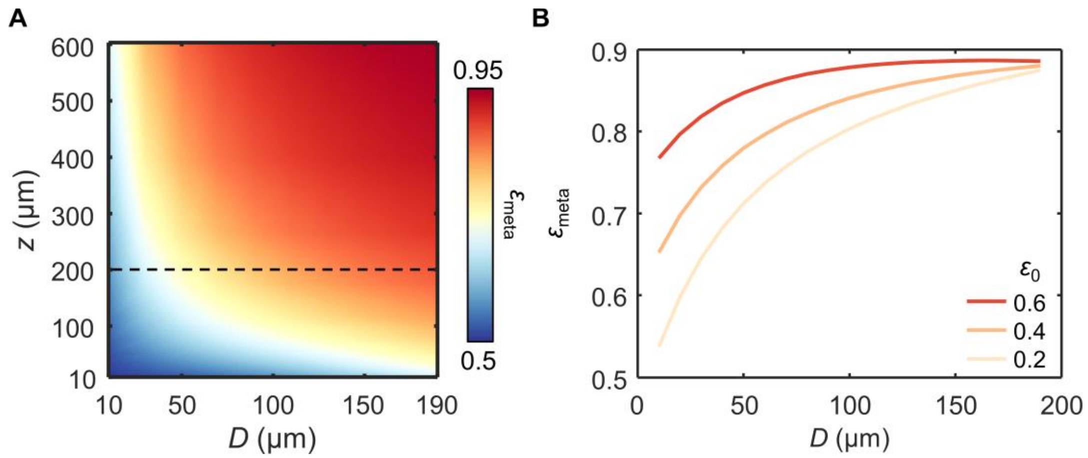

Fig. S2. Simulated effective emissivity of a hexagonal meta-atom with cylindrical microcavities. (**A**) Effect of the geometric dimensions (diameter *D* and depth *z*) on the effective emissivity ε_{meta} with $\varepsilon_0 = 0.25$ and $\varepsilon_1 = 0.50$. (**B**) Effect of the diameter *D* on the effective emissivity ε_{meta} with various initial emissivity ε_0 of the flat surface when $z = 200$ μm. The period is $P = 200$ μm.

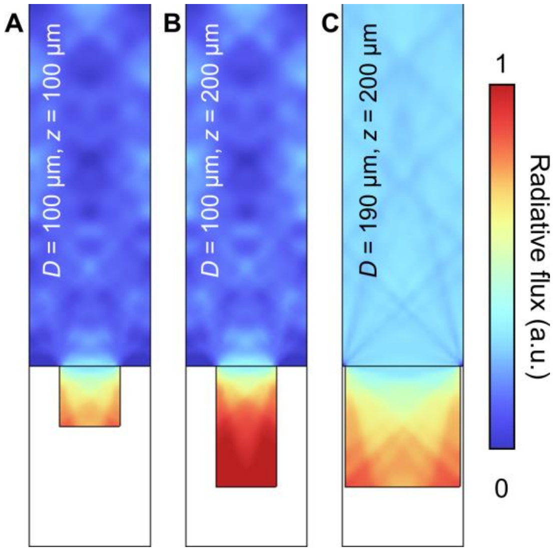

Fig. S3. Simulated 2D radiative flux of a single microcavity with various diameters *D* and depths *z*. (**A**) *D* =100 µm, *z* =100 µm, (**B**) *D* =100 µm, *z* =200 µm and (**C**) *D* =190 µm, *z* =200 µm.

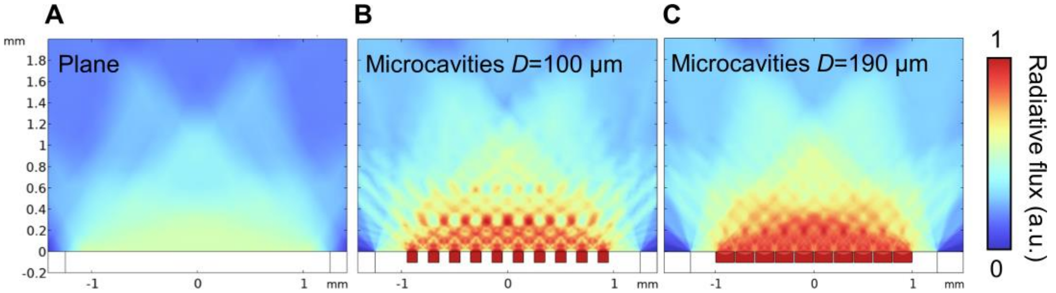

Fig. S4. Simulated 2D radiative flux. (**A**) The plane, (**B**) microcavities with *D* =100 μm and *z* = 100 μm, and (**C**) microcavities with *D* =190 μm and *z* = 100 μm. The period is *P* = 200 μm.

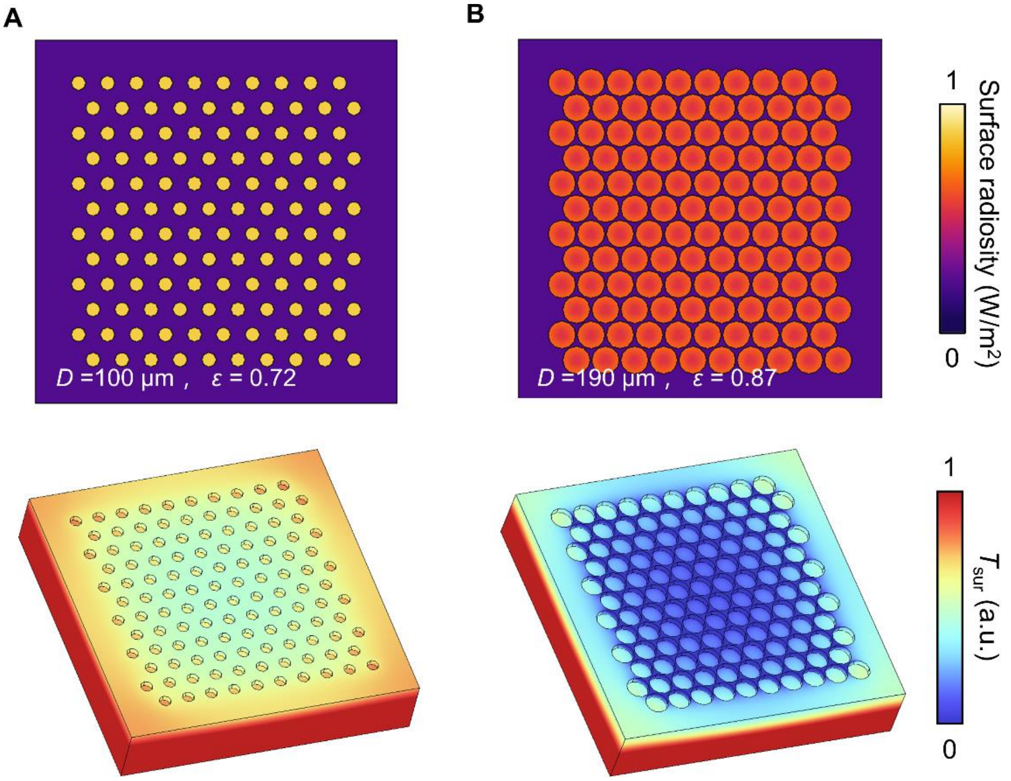

Fig. S5. Heat transfer simulation of surface radiosity (top) and surface temperature (bottom). Two diameters are D =100 µm for (**A**) and D =190 µm for (**B**). Depths and periods are constant at z = 200 µm, P = 200 µm.

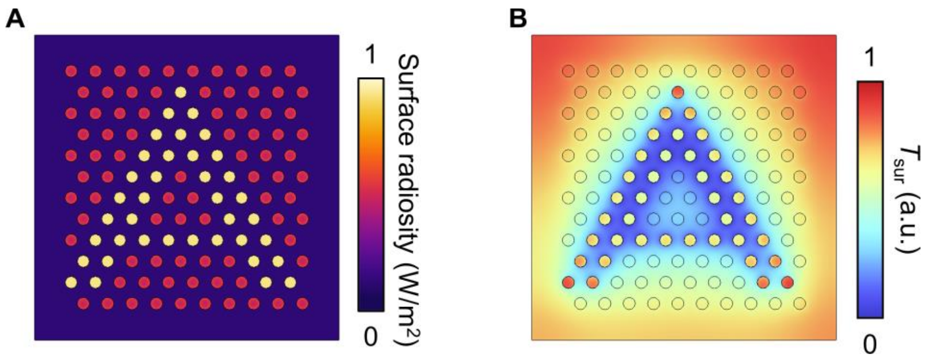

Fig. S6. Heat transfer simulation for dual-depth IR encryption. (**A**) Surface radiosity on the IR encrypted letter of “A” with dual-depth microcavities, such as z_1 =10 μm (dark circles) and z_2 =100 μm (light circles). (**B**) Surface temperature. The variation in surface radiosity generates a temperature gradient, producing an IR encrypted pattern.

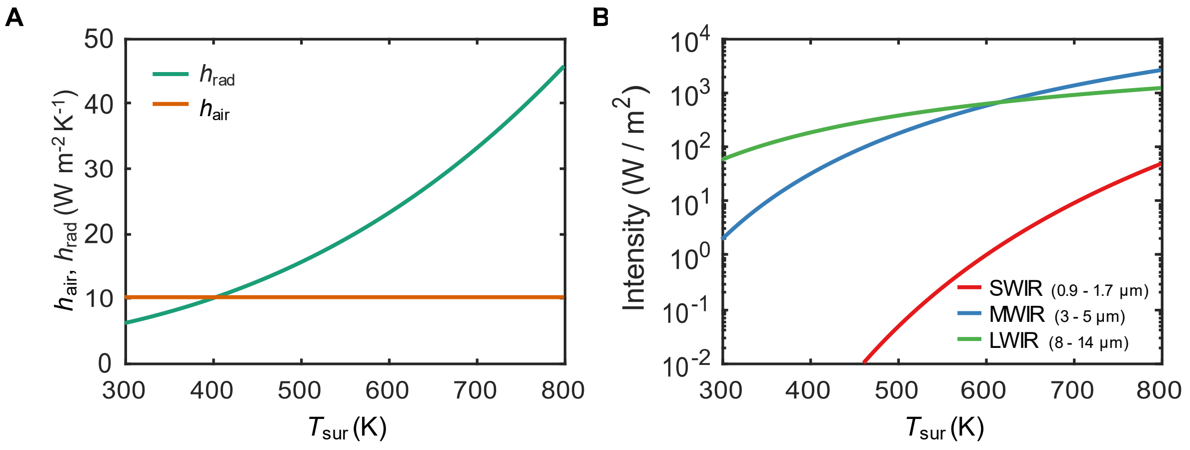

Fig. S7. Radiative efficiency of an ideal blackbody. (**A**) Effect of the surface temperature on convective coefficient and radiative coefficient. (**B**) Effect of the surface temperature on blackbody radiative intensity in three bands: short-wave infrared (SWIR), mid-wave infrared (MWIR) and long-wave infrared (LWIR), respectively.

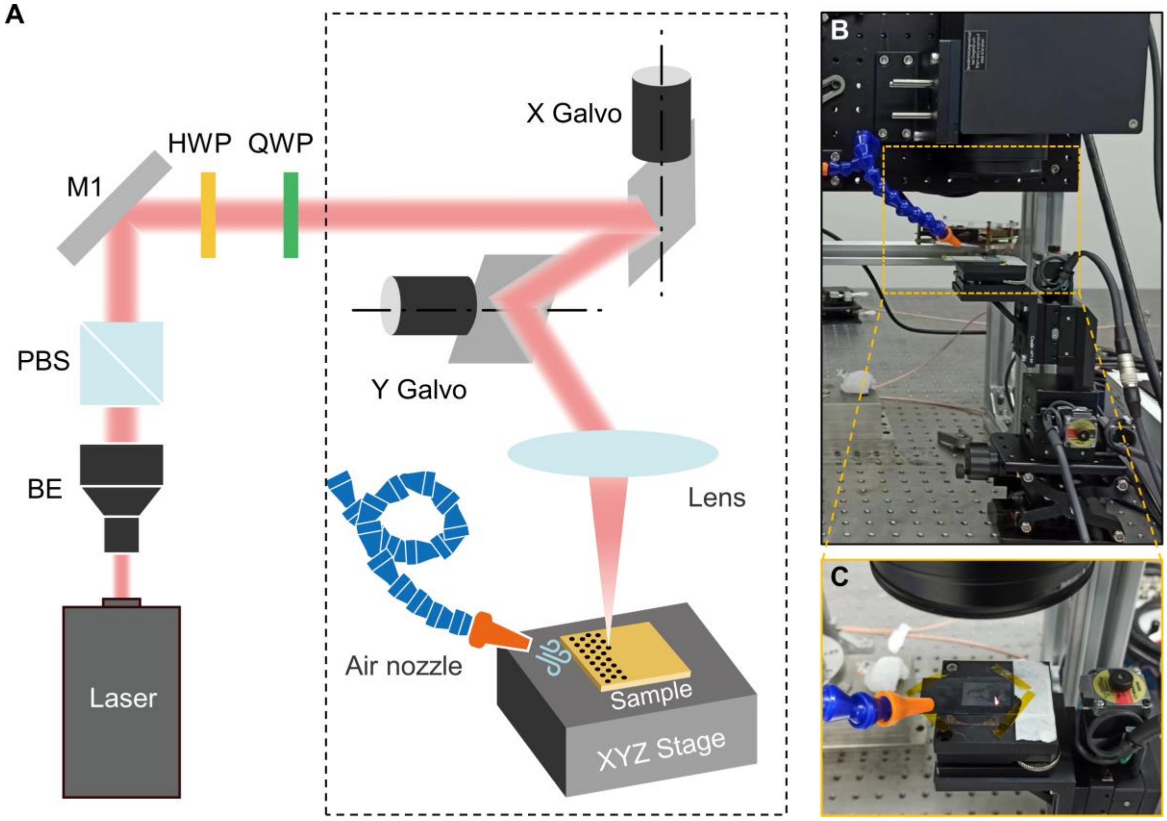

Fig. S8. Setup of the laser fabrication system. (**A**) Optical configuration. (**B)** Photographs. (**C**) Zoomed view.

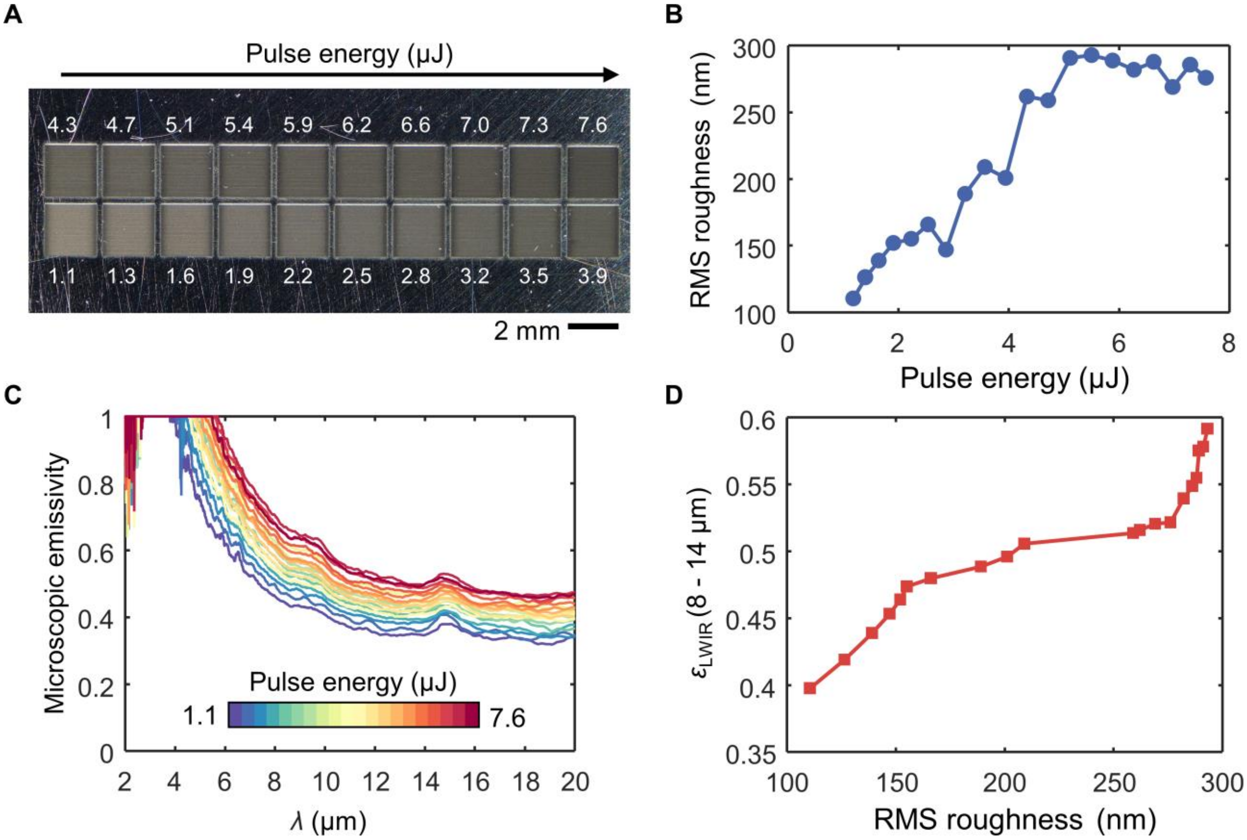

Fig. S9. Experiments of laser processing on polished titanium. (**A**) Optical image of the laser-treated areas with various pulse energies, constant velocity v = 600 mm/s and constant hatching pitch d_{H} = 2.5 μm. (**B**) Effect of the pulse energy on the root-mean-square (RMS) roughness. (**C**) Microscopic emissivity is measured using the sample itself as a heat source and a standard blackbody as a reference. Any measured emissivity that exceeded 1 is truncated to 1 during data processing. (**D**) Effect of RMS roughness on the average emissivity in the LWIR range of 8 μm to 14 μm.

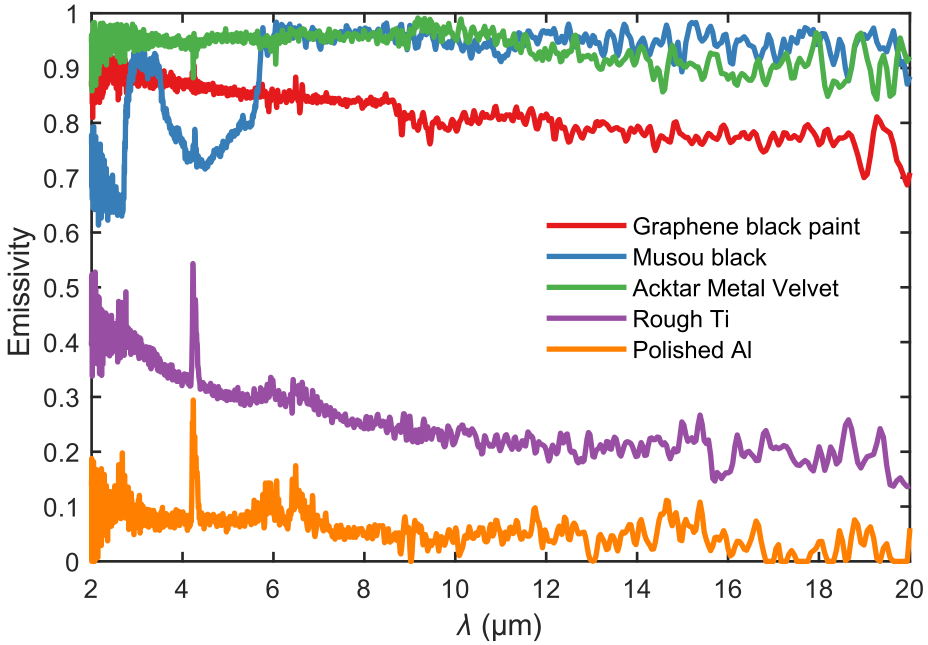

Fig. S10. Hemispheric emissivity of various samples. The average values in different wavelength ranges are indicated in table S2.

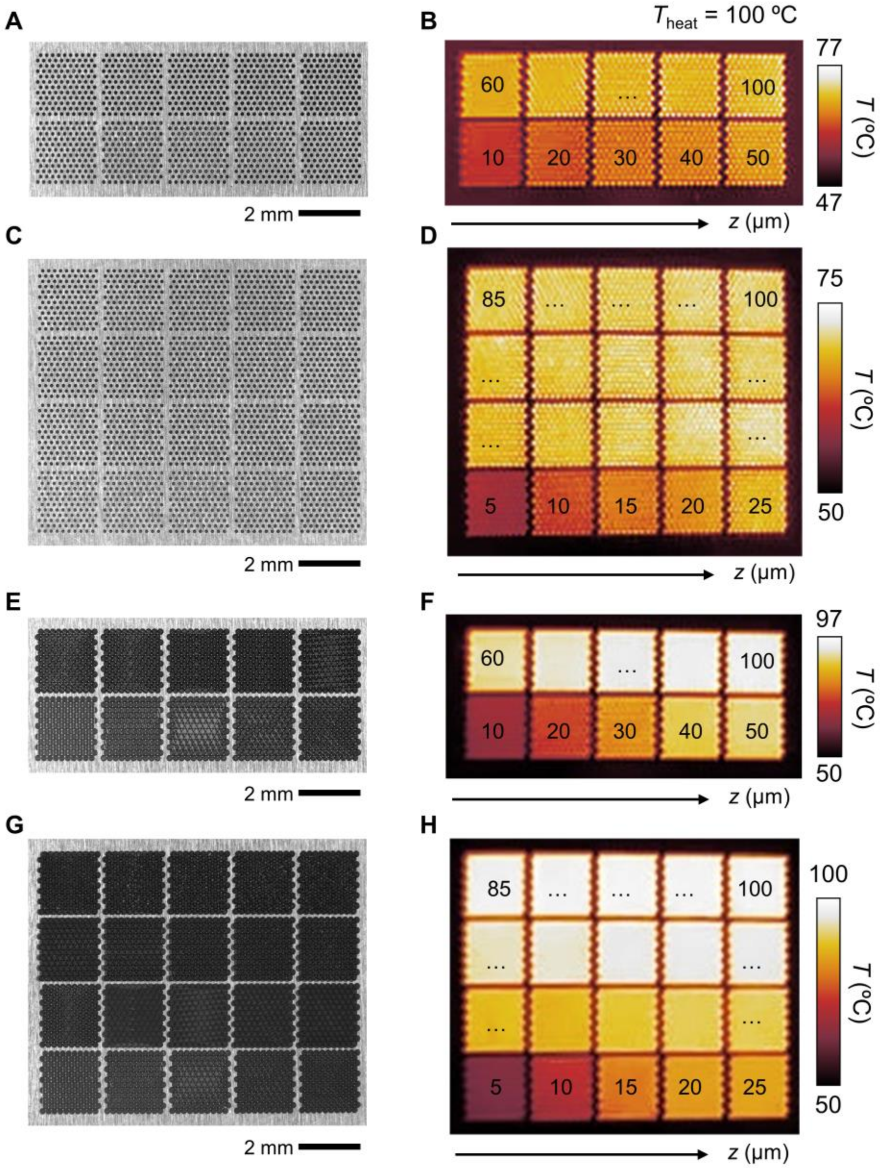

Fig. S11. Visible (left) and IR (right) images of titanium microcavities with different depths *z* = 10-100 µm, constant period *P* = 200 µm. The diameters are *D* = 100 µm for (**A** to **D**) and *D* = 190 µm for (**E** to **H**). Inserted numbers represent the values of depth *z*.

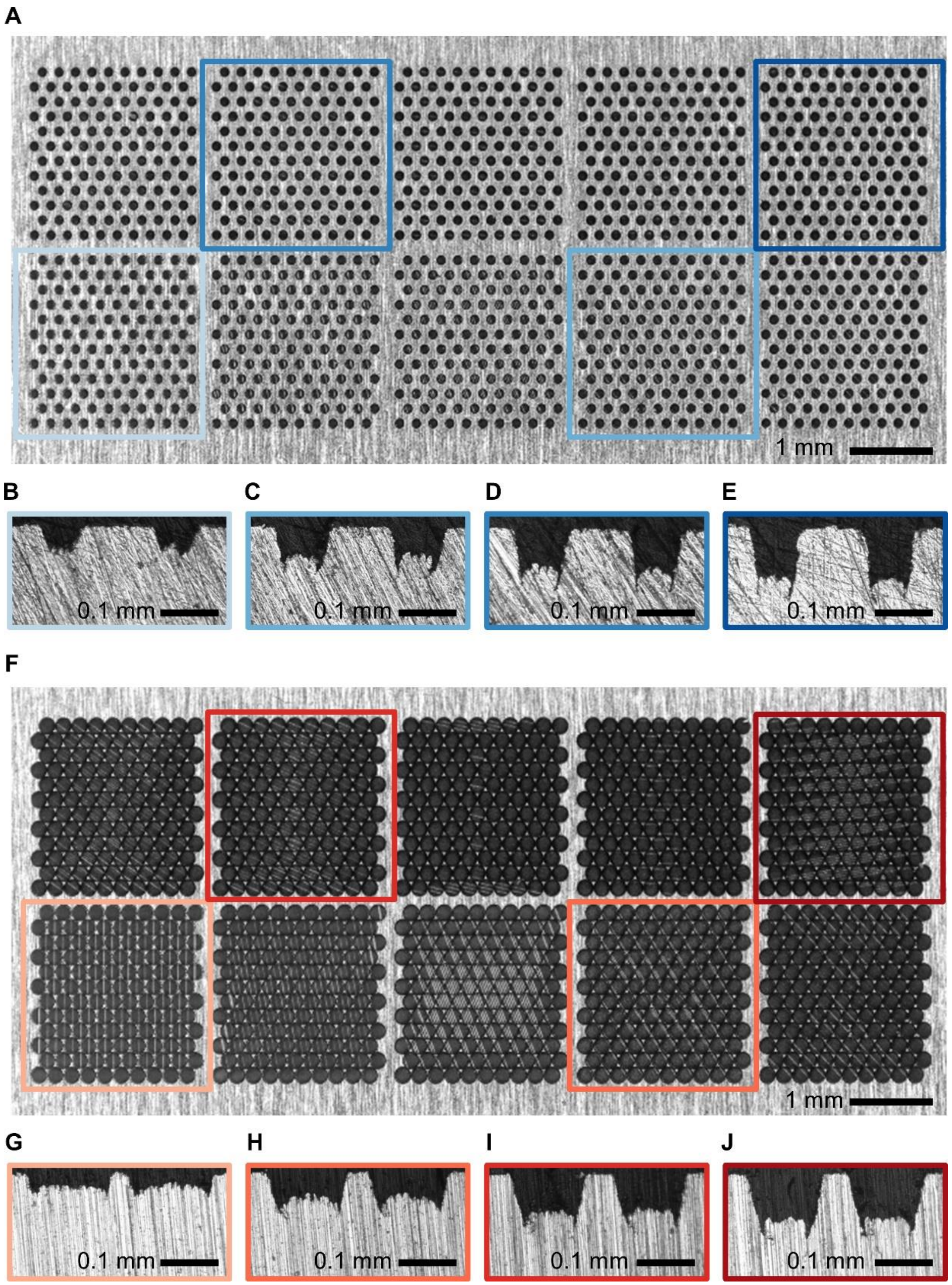

Fig. S12. Visible image of titanium microcavities with different depths z = 10-100 μm, constant period P = 200 μm. The diameters are D = 100 μm for (**A**) and D = 190 μm for (**F**). (**B** to **E**) Cross-sectional views in (A). (**G** to **J**) Cross-sectional views in (F). The IR images of (A, F) are indicated in Fig. 2D, E.

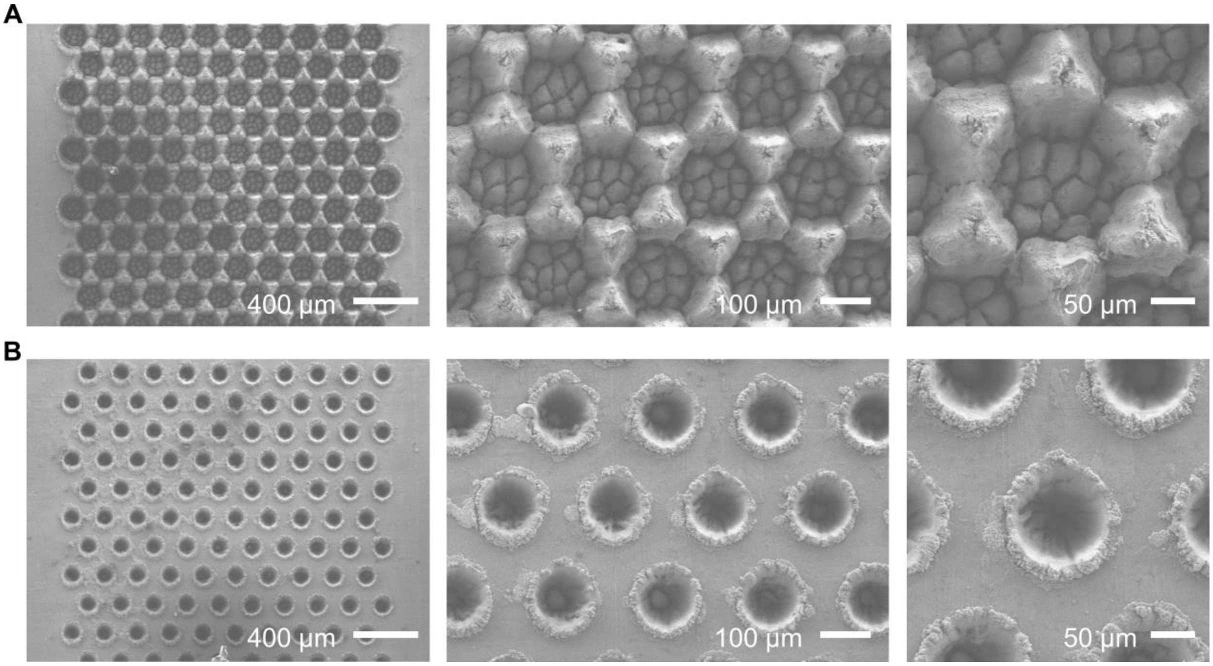

Fig. S13. SEM images of the titanium microcavities. The period is P = 200 μm, depth is z = 100 μm, and two diameters are D = 190 μm for (**A**) and D = 100 μm for (**B**).

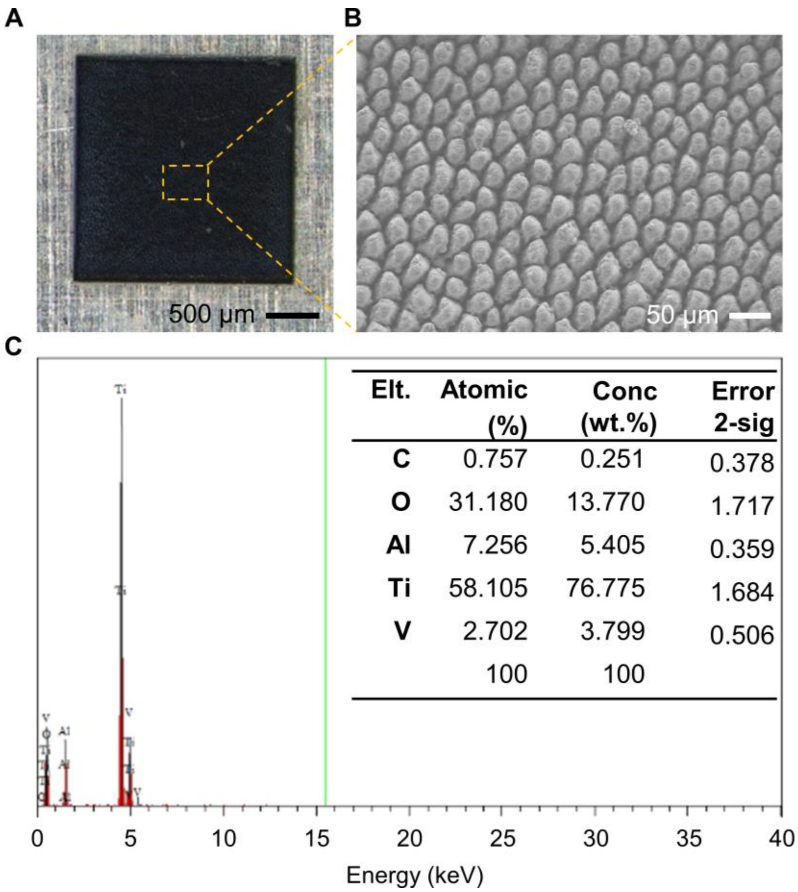

Elt.	Atomic (%)	Conc (wt.%)	Error 2-sig
C	0.757	0.251	0.378
O	31.180	13.770	1.717
Al	7.256	5.405	0.359
Ti	58.105	76.775	1.684
V	2.702	3.799	0.506
	100	100	

Fig. S14. Laser-induced black microstructures on titanium. (**A**) Optical image. (**B**) SEM image. (**C**) Energy dispersive X-ray (EDX) analysis. Inset is a table of elemental composition.

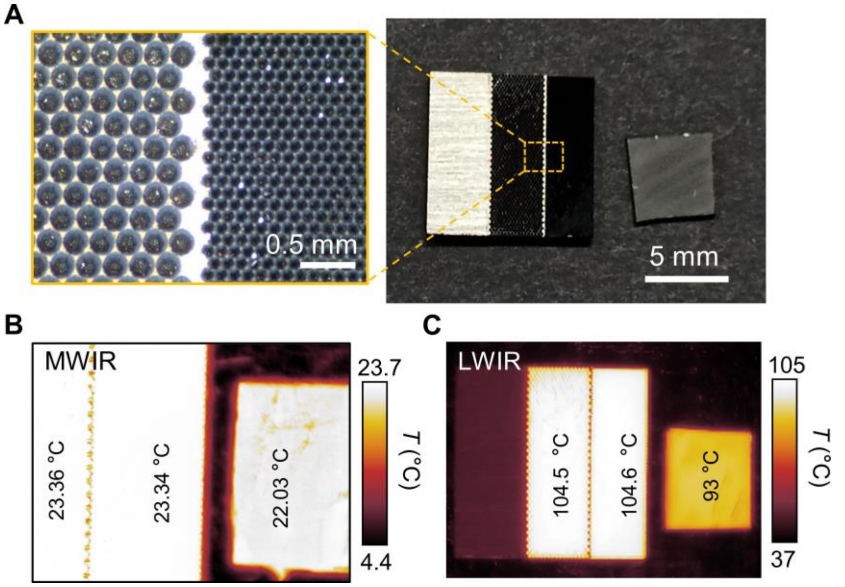

Fig. S15. Titanium microcavities and the graphene black paint (*45*)**.** (**A**) Visible, (**B**) MWIR and (**C**) LWIR images. The zoomed view of (A) indicates the microcavities with *D* = 180 µm, *P* = 200 µm for left and *D* = 90 µm, *P* = 100 µm for right. MWIR image was captured the samples at room temperature of 23 ºC while LWIR image was detected the samples at high temperature of 100 ºC.

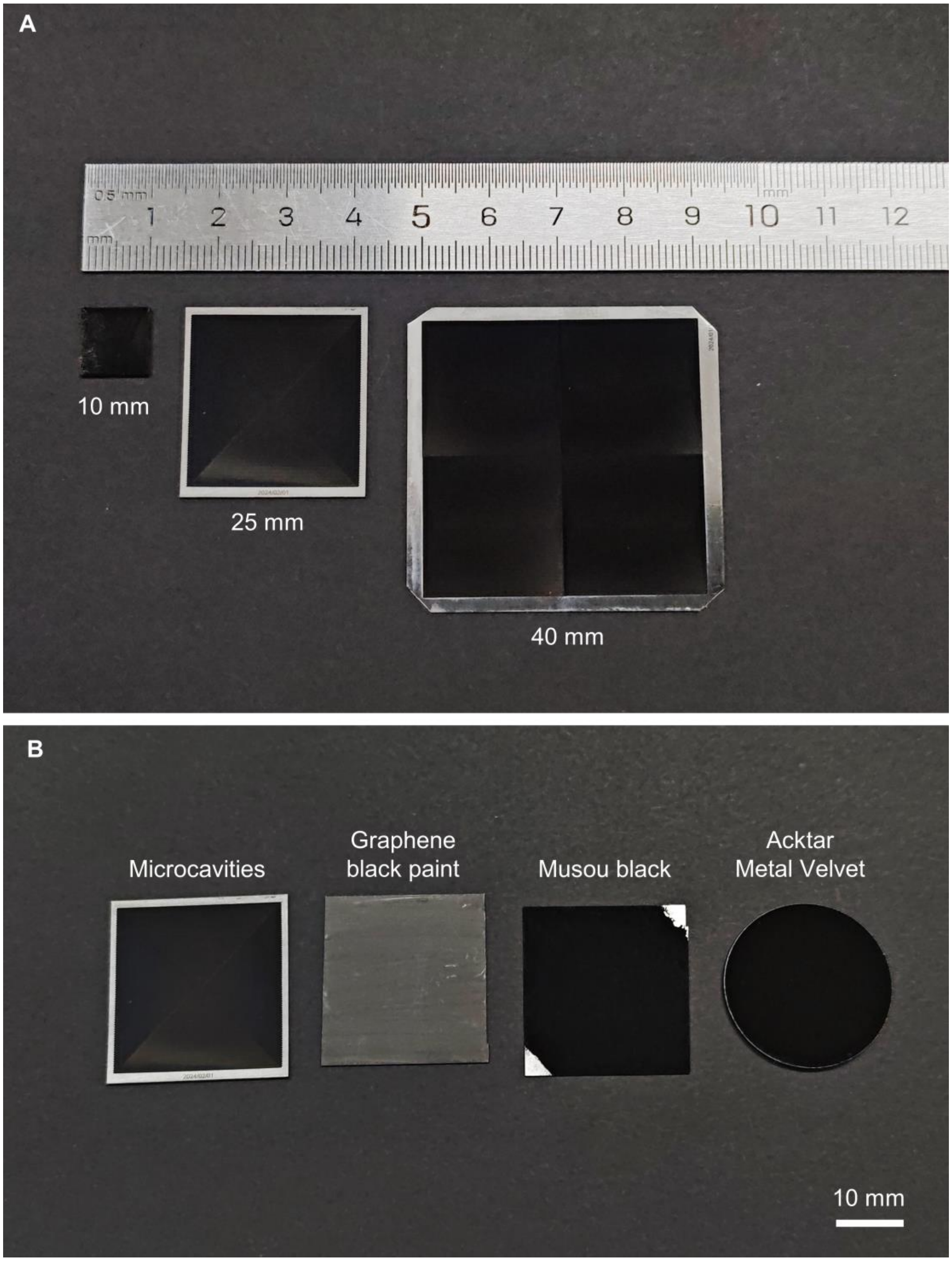

Fig. S16. Photographs of titanium microcavities. (**A**) Scalability of laser-printed microcavities with a maximum size up to 40 mm × 40 mm. All microcavities with P = 200 μm and D = 180 μm are fabricated on titanium sheets. (**B**) Photograph of microcavities and three blackbody coatings including graphene black paint (*45*), Musou black (*47*) and Acktar Metal Velvet (*46*).

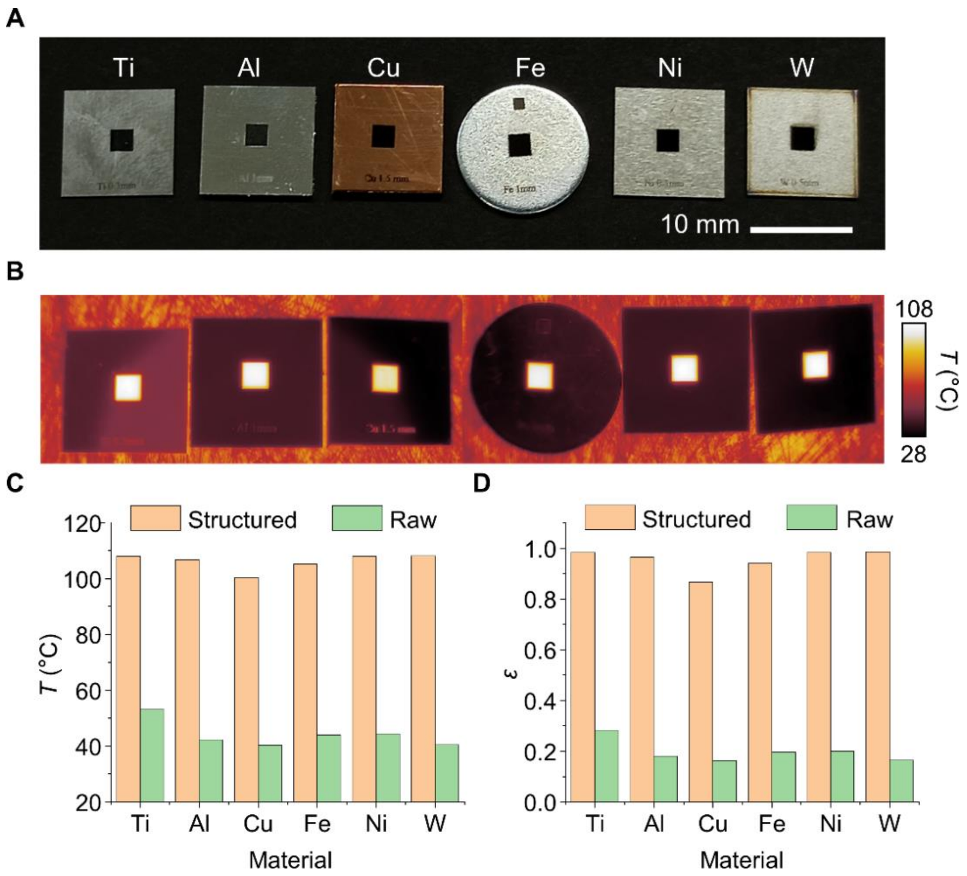

Fig. S17. Applicability of blackbody microcavities with *P* = 100 µm and *D* = 90 µm on various materials. (**A**) Visible image. (**B**) IR image. (**C**) Radiative temperature of structured and raw surfaces. (**D**) Calculated Emissivity of structured and raw surfaces.

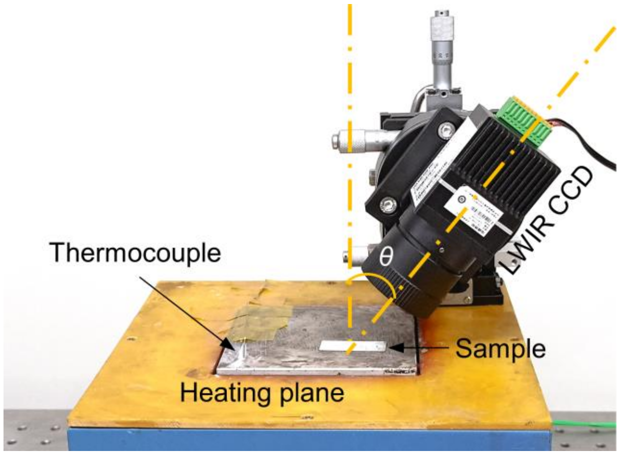

Fig. S18. Experimental setup of the IR imaging system with a tunable titled angle *θ*. A K-type thermocouple is employed to measure the surface temperature of the heating plane.

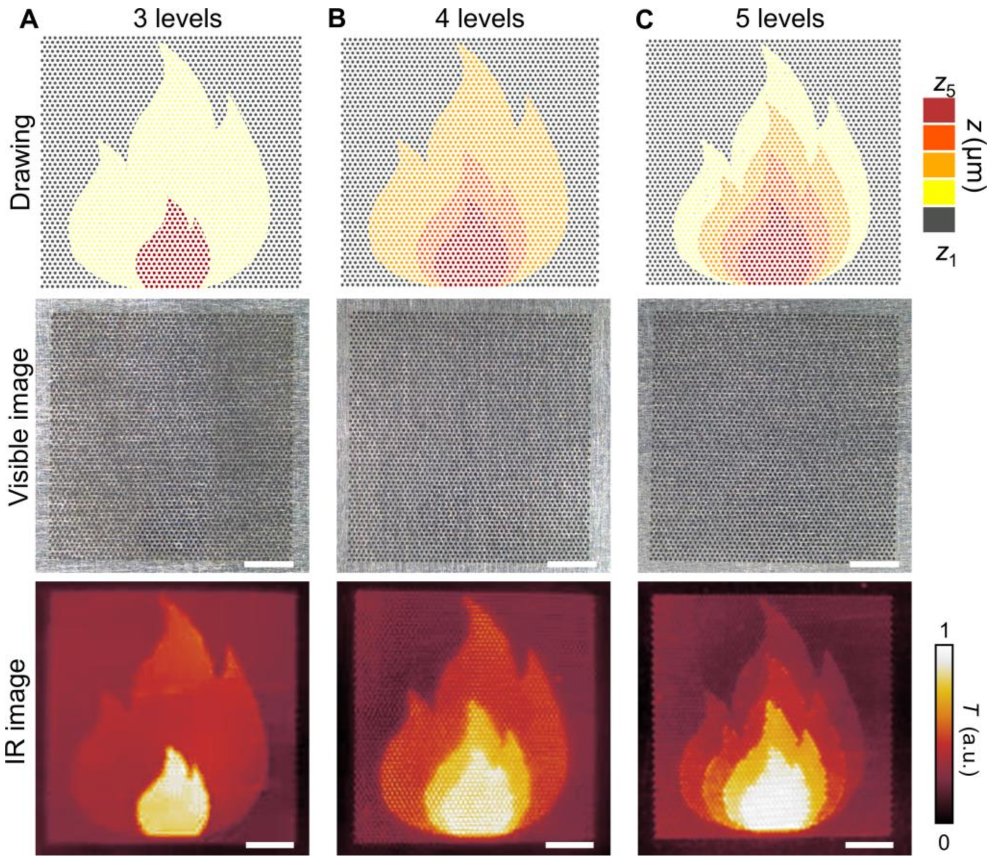

Fig. S19. IR encrypted pattens with multi-depth encoded microcavities. Drawing (top), visible images (middle) and IR images (bottom) of the multi-gradient fires created by the microcavities with a constant period P = 200 μm, diameter D = 100 μm and various depth z (e.g., from z_1 to z_5 in 5 levels). (**A**) 3 levels, (**B**) 4 levels, (**C**) 5 levels. Scale bars are 2 mm.

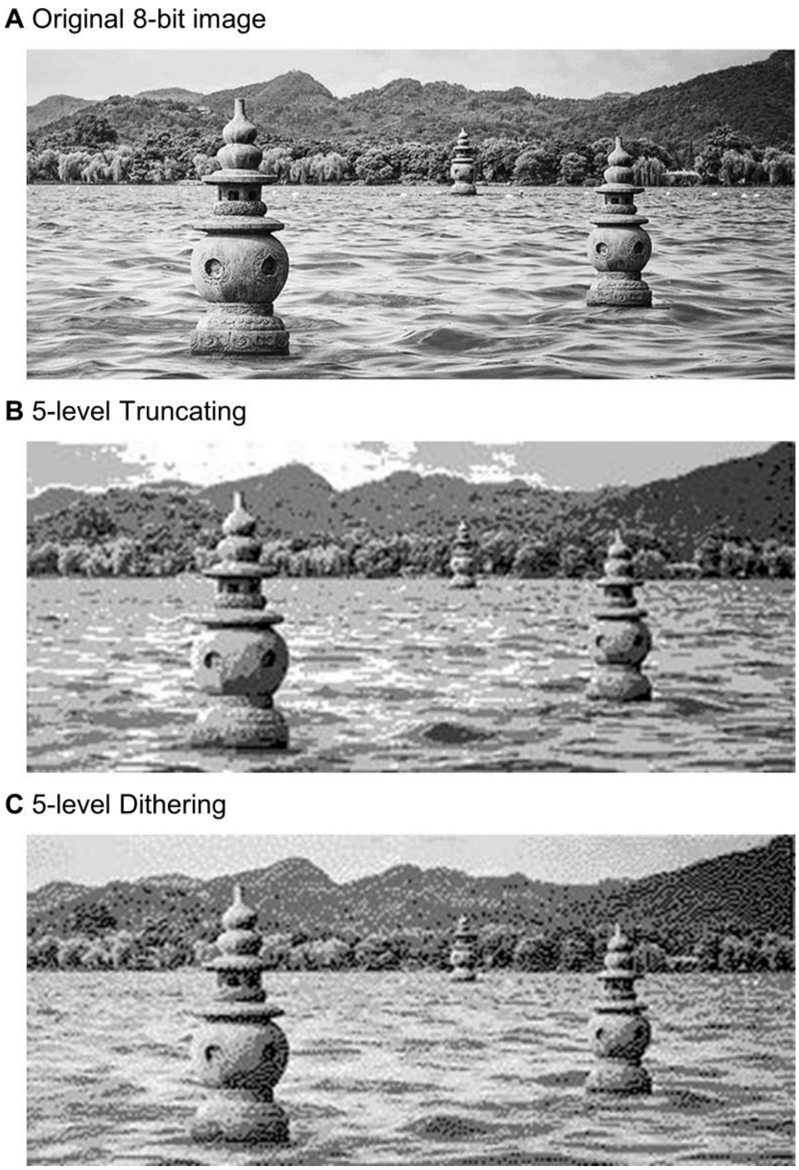

Fig. S20. Comparison of grayscale images with difference rendering. (**A**) Original 8-bit image. (**B**) 5-level Truncated image exhibiting pronounced color banding in the sky region. (**C**) 5-level Dithered image without distinct color banding.

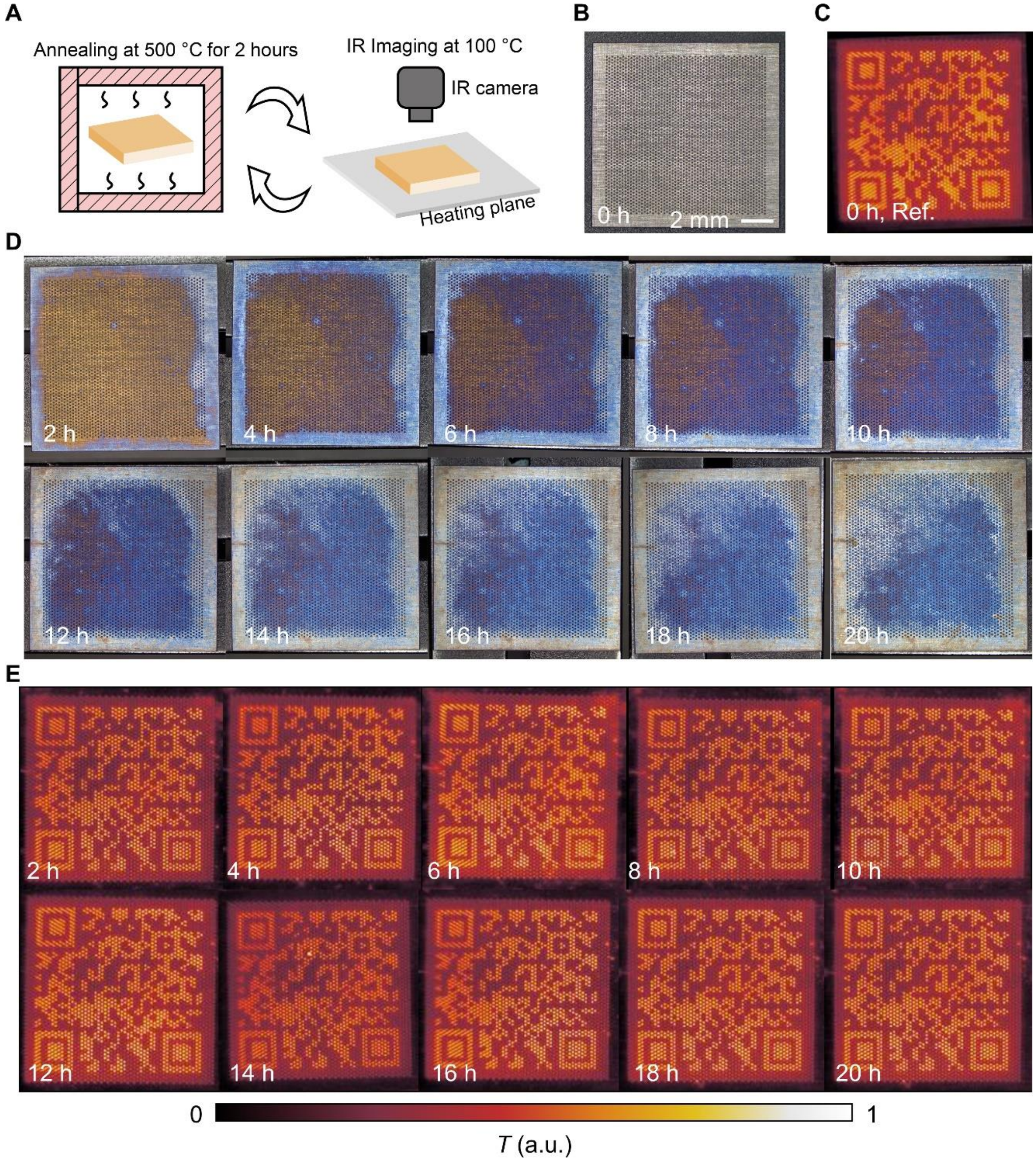

Fig. S21. Variation of the titanium microcavities after annealing at 500 ºC for several hours in steps of 2 hours. (**A**) Schematic of the experimental procedures. (**B**, **D**) Visible images of the microcavities before and after annealing, respectively. (**C**, **E**) IR images of the microcavities before and after annealing, respectively. The original IR image (C) is selected as the reference to calculate the Peak signal-to-noise ratio (PSNR). Inserted numbers represent total accumulated time for annealing. The PSNR of IR images are summarized in Fig. 3H.

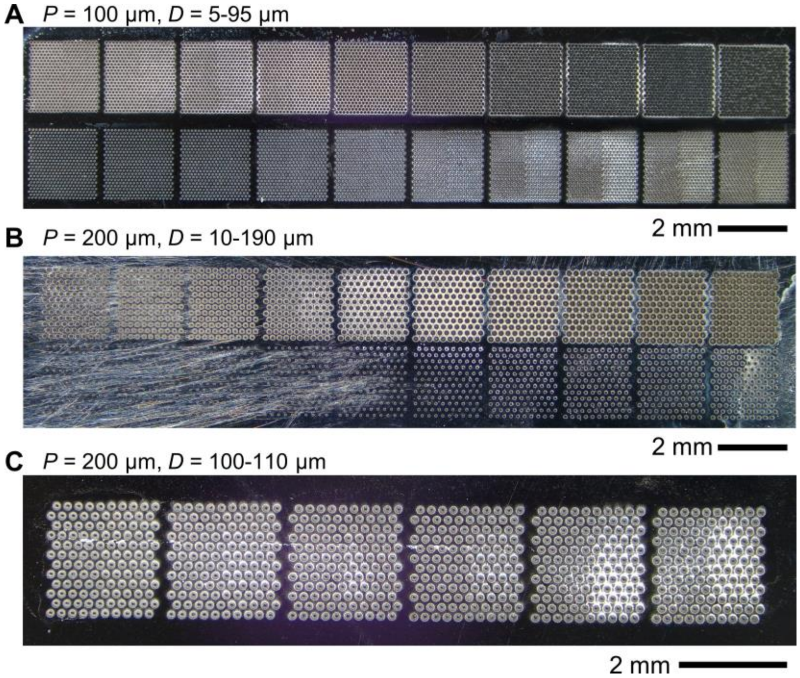

Fig. S22. Visible images of aluminum microcavities with various diameters *D* and periods *P*. (**A**) *P* = 100 µm, *D* = 5-95 µm. (**B**) *P* = 200 µm, *D* = 10-190 µm. (**C**) *P* = 200 µm, *D* = 100-110 µm. The IR images are indicated in Fig. 4A, C.

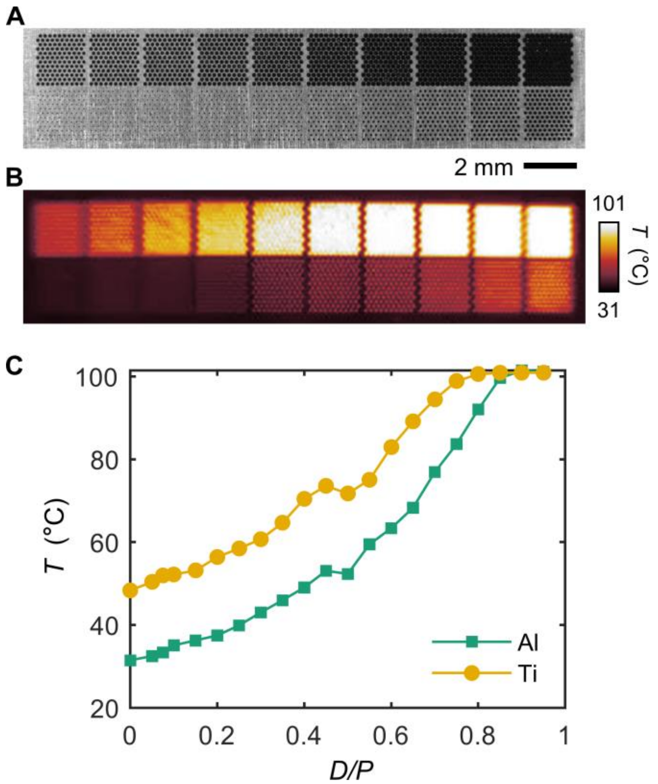

Fig. S23. Grayscale emissivity on titanium microcavities with a constant period *P* = 200 µm and various diameters *D*. (**A**) Visible image. (**B**) IR image. (**C**) Comparison of the radiative temperature between titanium microcavities with rough initial surface (R_q = 269 nm) and aluminum microcavities with smooth initial surface (R_q = 2.4 nm). The data of aluminum microcavities are collected from Fig. 4A.

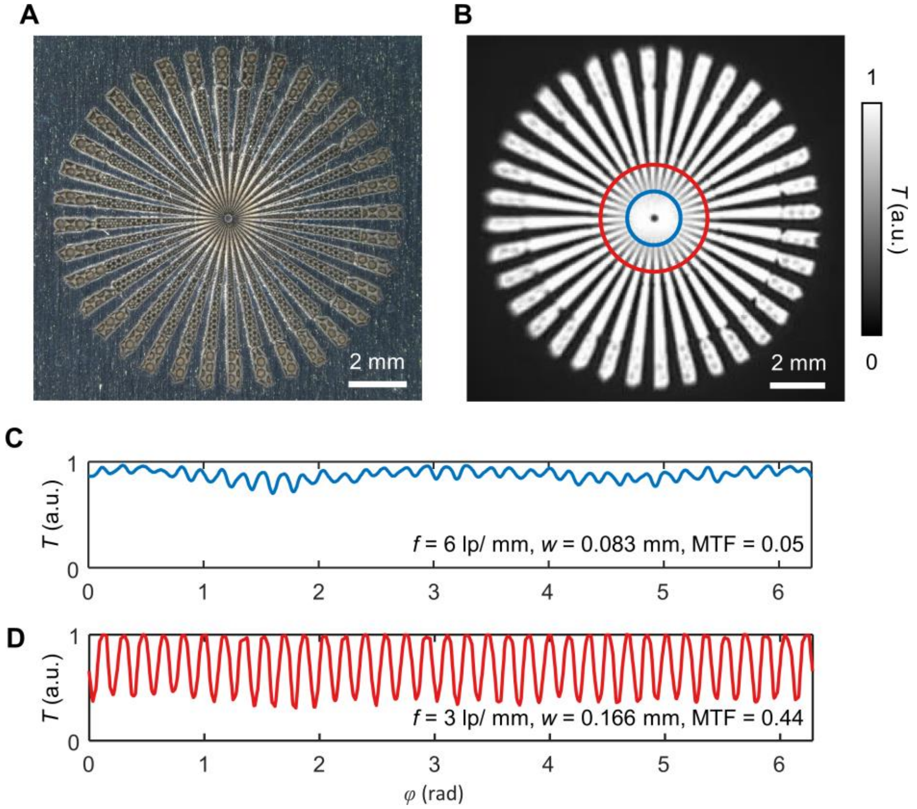

Fig. S24. Resolution star target (Siemens star) created by the blackbody microcavities on a polished aluminum sheet. D_o = 12 mm (f_{min} = 0.955 lp/mm) is for the outer edge and D_i = 0.286 mm (f_{max} = 40 lp/mm) is for the inner edge. (**A**) Visible image. (**B**) IR image. (**C**, **D**) The azimuthal distributions of radiative temperature are extracted from the IR image at f = 6 lp/mm and f = 3 lp/mm, respectively.

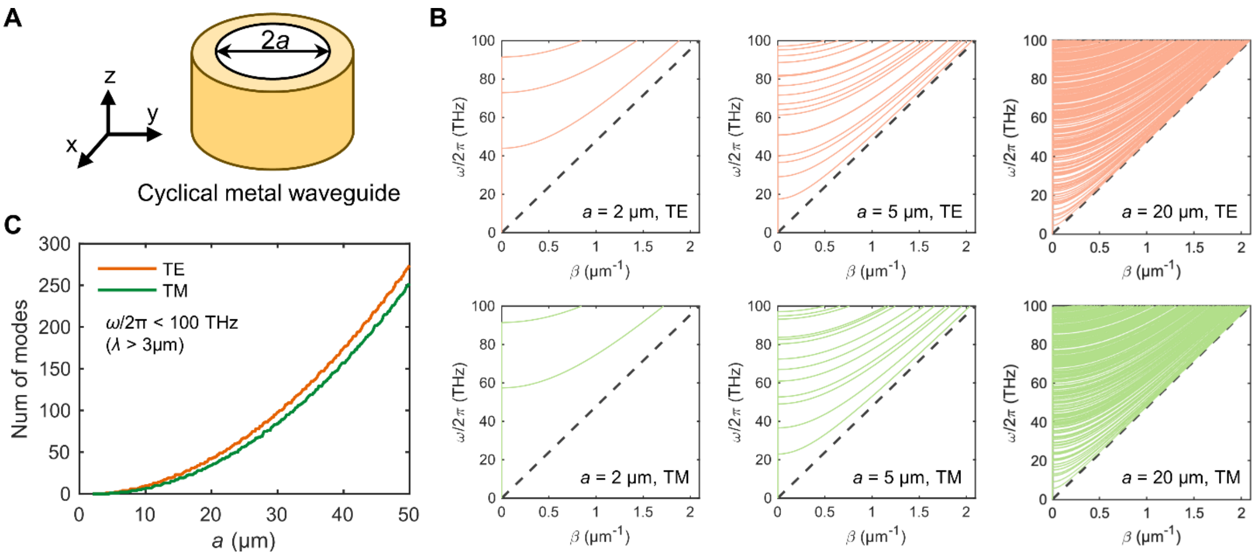

Fig. S25. Dispersion analysis of metal microcavities. (**A**) Schematic of cylindrical metal waveguide with the radius *a*, which is the ideal shape of the blackbody microcavities. (**B**) Dispersion curves of cylindrical metal waveguide with various radius *a* when propagating TE modes (top) and TM modes (bottom). The dashed line is the light line. (**C**) Number of waveguide modes as a function of the radius *a* in frequency range $\omega/2\pi < 100$ THz.

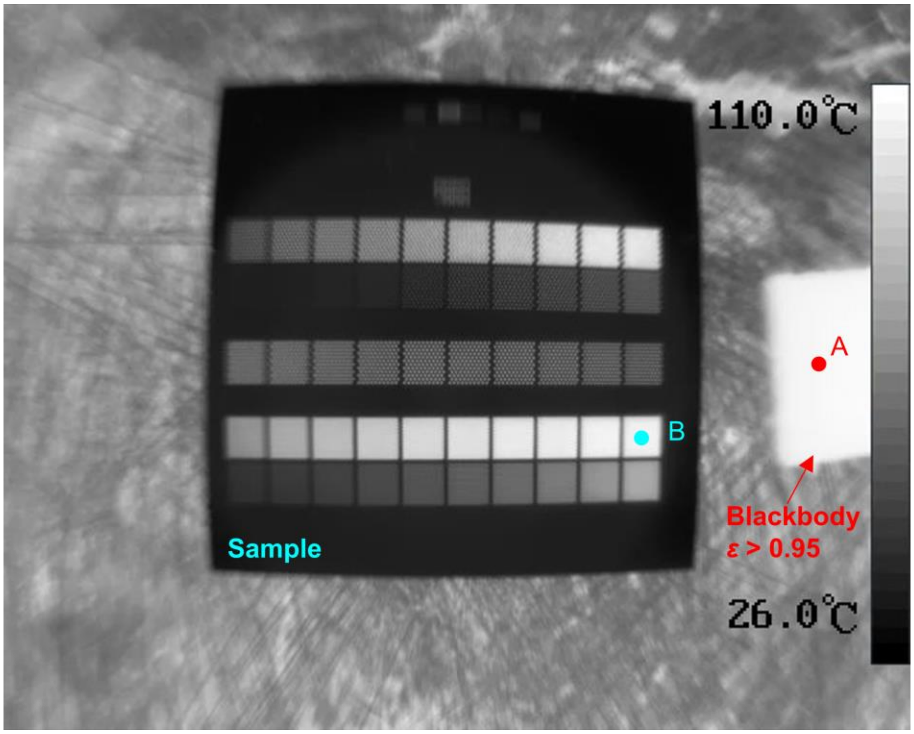

Fig. S26. Calibration of the LWIR image using a standard blackbody (ε > 0.95) as reference. Point A is located on the blackbody. Point B is located on the Sample. The temperature at point A match with the surface temperature, which is measured using a K-type thermocouple. Then, the calibrated LWIR image can be obtained. If the blackbody can be removed, the LWIR images of the specified sample can still be corrected using the calibrated temperature at point B as a reference. However, it is necessary to repeat the above steps whenever the heating temperature or the sample is changed.

Table S1. Fabrication parameters for various materials.

Material	Pulse energy E_p (μJ)	Velocity v (mm/s)	Hatching pitch d_H (μm)	Depth resolution Δz (μm)
Ti (Ti-6Al-4V)	4.32	650	2.5	7.3
Ti (Ti-6Al-4V)	0.62	900	3.0	2.5
Al (5052)	3.09	800	3.5	6.0
Fe (SECC)	4.32	650	2.5	9.7
Cu (C11000)	6.86	650	2.5	18.9
W (W1)	6.86	650	2.5	14.5
Ni (Ni200)	5.62	650	2.5	18.5

Table S2. Hemispheric emissivity averaged over different wavelength ranges.

Sample	ε_{IR} (2.5-20 µm)	ε_{MWIR} (3-5 µm)	ε_{LWIR} (8-14 µm)
**Ti Microcavities**	**0.9390**	**0.9500**	**0.9190**
Graphene black paint	0.8546	0.8740	0.8078
Musou black	0.8548	0.8176	0.9526
Acktar Metal Velvet	0.9483	0.9487	0.9509
Rough Ti	0.3221	0.3528	0.2273
Polished Al	0.0781	0.0843	0.0470

Table S3. Comparison of state-of-the-art IR radiative devices

Material	Fabrication	Functionalities	Emissivity	Res.	Max. size	Ref.
Cu	Laser ablation, Thermal oxidation	Broadband absorber	0.87 @ 5-25 µm	-	25 mm × 25 mm	(*52*)
GST film	Laser patterning	IR patterns	0.1-07 @ 3 µm<br>0.1-0.75 @ 8 µm	~ 0.6 µm	0.9 mm × 1.3 mm	(*53*)
GST film	Laser lithography	IR patterns	0.26-0.77 @ 7.5-13 µm	~ 2 µm	4-inch wafer	(*54*)
Au, SiO_2	Electron beam lithography	Chiral radiation	0.2-0.8 @ 7µm	3 µm	5 mm × 5 mm	(*43*)
Polymer film	Spin coating, Mechanical deformation	IR patterns	0.39-0.56 @ 7.5-14 µm	~ 5 mm	>50 mm × 50 mm	(*55*)
**Ti-6Al-4V**	**Hierarchical laser writing**	**Broadband absorber, IR patterns**	**0.94 @ 0.25-20 µm**<br>**0.23-0.92 @ 8-14 µm**	**< 20 µm**	**40 mm × 40 mm**	**This work**
**Al**	**Hierarchical laser writing**	**IR patterns**	**0.04-0.92 @ 8-14 µm**	**< 20 µm**	**40 mm × 40 mm**	**This work**

Movie S1. LWIR video of the QR code sample and resolution strip targets (f = 2.5 lp/mm and f = 5 lp/mm) during the heating process. The temperature increases from 25°C to 100°C. This data is used to calculate the PSNR-temperature relationship, as shown in Fig. 3G.

REFERENCES AND NOTES

1. V. Sapritsky, A. Prokhorov, Elements of blackbodies design, in *Blackbody Radiometry: Volume 1: Fundamentals*, V. Sapritsky, A. Prokhorov, Eds. (Springer, 2020), pp. 251–310.

2. E. W. Treuenfels, Emissivity of isothermal cavities. *J. Opt. Soc. Am.* **53**, 1162–1171 (1963).

3. Z. Zhang, M. Chen, L. Zhang, H. Li, H. Huang, Z. Zhang, P. Yu, Y. Niu, S. Gao, C. Wang, J. Jiang, A straightforward spectral emissivity estimating method based on constructing random rough surfaces. *Light Sci. Appl.* **12**, 266 (2023).

4. K. Mizuno, J. Ishii, H. Kishida, Y. Hayamizu, S. Yasuda, D. N. Futaba, M. Yumura, K. Hata, A black body absorber from vertically aligned single-walled carbon nanotubes. *Proc. Natl. Acad. Sci. U.S.A.* **106**, 6044–6047 (2009).

5. J. Lehman, A. Sanders, L. Hanssen, B. Wilthan, J. Zeng, C. Jensen, Very black infrared detector from vertically aligned carbon nanotubes and electric-field poling of lithium tantalate. *Nano Lett.* **10**, 3261–3266 (2010).

6. K. Amemiya, H. Koshikawa, M. Imbe, T. Yamaki, H. Shitomi, Perfect blackbody sheets from nano-precision microtextured elastomers for light and thermal radiation management. *J. Mater. Chem. C* **7**, 5418–5425 (2019).

7. Y. Hou, D. Myung, J. K. Park, J. Min, H.-R. Lee, A. A. El-Aty, M.-G. Lee, A review of characterization and modelling approaches for sheet metal forming of lightweight metallic materials. *Materials* **16**, 836 (2023).

8. Z. Liao, A. la Monaca, J. Murray, A. Speidel, D. Ushmaev, A. Clare, D. Axinte, R. M'Saoubi, Surface integrity in metal machining—Part I: Fundamentals of surface characteristics and formation mechanisms. *Int. J. Mach. Tool Manuf.* **162**, 103687 (2021).

9. A. V. Prokhorov, L. M. Hanssen, S. N. Mekhontsev, Radiation properties of IR calibrators with V-grooved surfaces, in *Thermosense XXVIII* (SPIE, 2006), vol. 6205, pp. 28–36.

10. F. Olschewski, A. Ebersoldt, F. Friedl-Vallon, B. Gutschwager, J. Hollandt, A. Kleinert, C. Monte, C. Piesch, P. Preusse, C. Rolf, P. Steffens, R. Koppmann, The in-flight blackbody calibration system for the GLORIA interferometer on board an airborne research platform. *Atmos. Meas. Tech.* **6**, 3067–3082 (2013).

11. F. Olschewski, C. Monte, A. Adibekyan, M. Reiniger, B. Gutschwager, J. Hollandt, R. Koppmann, A large-area blackbody for in-flight calibration of an infrared interferometer deployed on board a long-duration balloon for stratospheric research. *Atmos. Meas. Tech.* **11**, 4757–4762 (2018).

12. A. R. Karoli, J. R. Hickey, R. E. Nelson, An absolute calibration source for laboratory and satellite infrared spectrometers. *Appl. Optics* **6**, 1183–1188 (1967).

13. Y. Zhou, Z. Qin, Z. Liang, D. Meng, H. Xu, D. R. Smith, Y. Liu, Ultra-broadband metamaterial absorbers from long to very long infrared regime. *Light Sci. Appl.* **10**, 138 (2021).

14. M. S. Ergoktas, A. Kecebas, K. Despotelis, S. Soleymani, G. Bakan, A. Kocabas, A. Principi, S. Rotter, S. K. Ozdemir, C. Kocabas, Localized thermal emission from topological interfaces. *Science* **384**, 1122–1126 (2024).

15. Z. Fan, T. Hwang, S. Lin, Y. Chen, Z. J. Wong, Directional thermal emission and display using pixelated non-imaging micro-optics. *Nat. Commun.* **15**, 4544 (2024).

16. J. Geng, L. Xu, W. Yan, L. Shi, M. Qiu, High-speed laser writing of structural colors for full-color inkless printing. *Nat. Commun.* **14**, 565 (2023).

17. C. Chen, C. Kuong Ng, F. Zhang, X. Xiong, B.-F. Ju, Y. Zhang, H. Nørgaard Hansen, Y.-L. Chen, Towards obtaining high-quality surfaces with nanometric finish by femtosecond laser ablation: A case study on coppers. *Opt. Laser Technol.* **155**, 108382 (2022).

18. X.-J. Wang, H.-H. Fang, Z.-Z. Li, D. Wang, H.-B. Sun, Laser manufacturing of spatial resolution approaching quantum limit. *Light Sci. Appl.* **13**, 6 (2024).

19. O. Deussen, T. Isenberg, Halftoning and stippling, in *Image and Video-Based Artistic Stylisation*, P. Rosin, J. Collomosse, Eds. (Springer, 2013), pp. 45–61.

20. S. C. Singh, M. ElKabbash, Z. Li, X. Li, B. Regmi, M. Madsen, S. A. Jalil, Z. Zhan, J. Zhang, C. Guo, Solar-trackable super-wicking black metal panel for photothermal water sanitation. *Nat. Sustain.* **3**, 938–946 (2020).

21. Y. Guo, K. Tsuda, S. Hosseini, Y. Murakami, A. Tricoli, J. Coventry, W. Lipiński, J. F. Torres, Scalable nano-architecture for stable near-blackbody solar absorption at high temperatures. *Nat. Commun.* **15**, 384 (2024).

22. H. Zhu, Q. Li, C. Zheng, Y. Hong, Z. Xu, H. Wang, W. Shen, S. Kaur, P. Ghosh, M. Qiu, High-temperature infrared camouflage with efficient thermal management. *Light Sci. Appl.* **9**, 60 (2020).

23. M. Pan, Y. Huang, Q. Li, H. Luo, H. Zhu, S. Kaur, M. Qiu, Multi-band middle-infrared-compatible camouflage with thermal management via simple photonic structures. *Nano Energy* **69**, 104449 (2020).

24. K.-T. Lin, X. Nian, K. Li, J. Han, N. Zheng, X. Lu, C. Guo, H. Lin, B. Jia, Highly efficient flexible structured metasurface by roll-to-roll printing for diurnal radiative cooling. *eLight* **3**, 22 (2023).

25. Z. Xu, Q. Li, K. Du, S. Long, Y. Yang, X. Cao, H. Luo, H. Zhu, P. Ghosh, W. Shen, M. Qiu, Spatially resolved dynamically reconfigurable multilevel control of thermal emission. *Laser Photonics Rev.* **14**, 1900162 (2020).

26. J. Hu, K. Xu, P. Huang, M. Wang, S. Xu, Q.-H. Wei, Hierarchical conical metasurfaces as ultra-broadband perfect absorbers from visible to far-infrared regime. *Adv. Funct. Mater.* **34**, 2309229 (2024).

27. F. Bao, X. Wang, S. H. Sureshbabu, G. Sreekumar, L. Yang, V. Aggarwal, V. N. Boddeti, Z. Jacob, Heat-assisted detection and ranging. *Nature* **619**, 743–748 (2023).

28. J. Wang, F. Yang, L. Xu, J. Huang, Omnithermal restructurable metasurfaces for both infrared-light illusion and visible-light similarity. *Phys. Rev. Appl.* **14**, 014008 (2020).

29. P. Jin, J. Liu, F. Yang, F. Marchesoni, J.-H. Jiang, J. Huang, In situ simulation of thermal reality. *Research* **6**, 0222 (2023).

30. J. Bonse, S. Gräf, Maxwell meets marangoni—A review of theories on laser-induced periodic surface structures. *Laser Photonics Rev.* **14**, 2000215 (2020).

31. J. Cheng, C.-s. Liu, S. Shang, D. Liu, W. Perrie, G. Dearden, K. Watkins, A review of ultrafast laser materials micromachining. *Opt. Laser Technol.* **46**, 88–102 (2013).

32. S. Lei, X. Zhao, X. Yu, A. Hu, S. Vukelic, M. B. G. Jun, H.-E. Joe, Y. L. Yao, Y. C. Shin, Ultrafast laser applications in manufacturing processes: A state-of-the-art review. *J. Manuf. Sci. Eng.* **142**, 031005 (2020).

33. S. A. Jalil, B. Lai, M. ElKabbash, J. Zhang, E. M. Garcell, S. Singh, C. Guo, Spectral absorption control of femtosecond laser-treated metals and application in solar-thermal devices. *Light Sci. Appl.* **9**, 14 (2020).

34. M. Park, L. Grbčić, P. Motameni, S. Song, A. Singh, D. Malagrino, M. Elzouka, P. H. Vahabi, A. Todeschini, W. A. De Jong, R. Prasher, V. Zorba, S. D. Lubner, Inverse design of photonic surfaces via high throughput femtosecond laser processing and tandem neural networks. *Adv. Sci.* **11**, 2401951 (2024).

35. Z. M. Zhang, Radiative properties of nanomaterials, in *Nano/Microscale Heat Transfer*, Z. M. Zhang, Ed. (Springer, 2020), pp. 497–622.

36. K.-T. Lin, H. Lin, T. Yang, B. Jia, Structured graphene metamaterial selective absorbers for high efficiency and omnidirectional solar thermal energy conversion. *Nat. Commun.* **11**, 1389 (2020).

37. L. Roberts, Picture coding using pseudo-random noise. *IRE Trans. Inf. Theory* **8**, 145–154 (1962).

38. M. Zhao, J. Wen, Q. Hu, X. Wei, Y.-W. Zhong, H. Ruan, M. Gu, A 3D nanoscale optical disk memory with petabit capacity. *Nature* **626**, 772–778 (2024).

39. D. K. Pradhan, D. C. Moore, G. Kim, Y. He, P. Musavigharavi, K.-H. Kim, N. Sharma, Z. Han, X. Du, V. S. Puli, E. A. Stach, W. Joshua Kennedy, N. R. Glavin, R. H. Olsson, D. Jariwala, A scalable ferroelectric non-volatile memory operating at 600°C. *Nat. Electron.* **7**, 348–355 (2024).

40. C.-L. Zhou, Z. Torbatian, S.-H. Yang, Y. Zhang, H.-L. Yi, M. Antezza, D. Novko, C.-W. Qiu, Unconventional thermophotonic charge density wave. *Phys. Rev. Lett.* **133**, 066902 (2024).

41. S. Yang, M. Liu, C. Zhao, S. Fan, C.-W. Qiu, Nonreciprocal thermal photonics. *Nat. Photonics* **18**, 412–424 (2024).

42. T. Liu, C. Guo, W. Li, S. Fan, Thermal photonics with broken symmetries. *eLight* **2**, 25 (2022).

43. X. Wang, T. Sentz, S. Bharadwaj, S. K. Ray, Y. Wang, D. Jiao, L. Qi, Z. Jacob, Observation of nonvanishing optical helicity in thermal radiation from symmetry-broken metasurfaces. *Sci. Adv.* **9**, eade4203 (2023).

44. J. M. Liu, Simple technique for measurements of pulsed Gaussian-beam spot sizes. *Opt. Lett.* **7**, 196–198 (1982).

45. Suzhou Tanfeng Graphene Technology, High-temperature-resistant oil-based graphene paint. https://www.graphenechina.com/.

46. Acktar, Metal velvet. https://acktar.com/black-coating-services/.

47. The Black Market, Koyo Orient Japan Co., Ltd., Musou black paint. https://the-black-market.com/zh.

48. G. Welsch, R. Boyer, E. W. Collings, *Materials Properties Handbook: Titanium Alloys* (ASM International, 1993).

49. G. Huang, A. R. Yengannagari, K. Matsumori, P. Patel, A. Datla, K. Trindade, E. Amarsanaa, T. Zhao, U. Köhler, D. Busko, B. S. Richards, Radiative cooling and indoor light management enabled by a transparent and self-cleaning polymer-based metamaterial. *Nat. Commun.* **15**, 3798 (2024).

50. M. Bauer, V. Volchkov, M. Hirsch, B. Schcölkopf, Automatic estimation of modulation transfer functions, in *2018 IEEE International Conference on Computational Photography (ICCP)* (IEEE, 2018), pp. 1–12.

51. C. Loebich, D. Wueller, B. Klingen, A. Jaeger, Digital camera resolution measurement using sinusoidal Siemens stars (SPIE, 2007), vol. 6502.

52. P. Fan, B. Bai, J. Long, D. Jiang, G. Jin, H. Zhang, M. Zhong, Broadband high-performance infrared antireflection nanowires facilely grown on ultrafast laser structured Cu surface. *Nano Lett.* **15**, 5988–5994 (2015).

53. Z. Xu, H. Luo, H. Zhu, Y. Hong, W. Shen, J. Ding, S. Kaur, P. Ghosh, M. Qiu, Q. Li, Nonvolatile optically reconfigurable radiative metasurface with visible tunability for anticounterfeiting. *Nano Lett.* **21**, 5269–5276 (2021).

54. Y. Kim, C. Kim, M. Lee, Parallel laser printing of a thermal emission pattern in a phase-change thin film cavity for infrared camouflage and security. *Laser Photonics Rev.* **16**, 2100545 (2022).

55. Y. Zhang, H. Zhu, S. An, W. Xing, B. Fu, P. Tao, W. Shang, J. Wu, M. D. Dickey, C. Song, T. Deng, Chameleon-inspired tunable multi-layered infrared-modulating system via stretchable liquid metal microdroplets in elastomer film. *Nat. Commun.* **15**, 5395 (2024).
